# Contextual Deprivation, Race and Ethnicity, and Income in Air Pollution and Cardiovascular Disease

**DOI:** 10.1001/jamanetworkopen.2024.29137

**Published:** 2024-08-19

**Authors:** Jiajun Luo, Andrew Craver, Zhihao Jin, Liang Zheng, Karen Kim, Tamar Polonsky, Christopher O. Olopade, Jayant M. Pinto, Habibul Ahsan, Briseis Aschebrook-Kilfoy

**Affiliations:** 1 Department of Public Health Sciences, Biological Science Division, The University of Chicago, Chicago, Illinois; 2 Institute for Population and Precision Health, Biological Science Division, The University of Chicago, Chicago, Illinois; 3 Gangarosa Department of Environmental Health, Rollins School of Public Health, Emory University, Atlanta, Georgia; 4 Department of Thyroid Surgery, the First Hospital Affiliated with Sun Yat-Sen University, Guangzhou, China; 5 Department of Medicine, Pennsylvania State College of Medicine, Hershey; 6 Department of Medicine, Biological Science Division, The University of Chicago, Chicago, Illinois; 7 Department of Family Medicine, Biological Science Division, The University of Chicago, Chicago, Illinois; 8 Department of Surgery, Biological Science Division, The University of Chicago, Chicago, Illinois

## Abstract

**Importance:**

Socioeconomically disadvantaged subpopulations are more vulnerable to fine particulate matter (PM_2.5_) exposure. However, as prior studies focused on individual-level socioeconomic characteristics, how contextual deprivation modifies the association of PM_2.5_ exposure with cardiovascular health remains unclear.

**Objective:**

To assess disparities in PM_2.5_ exposure association with cardiovascular disease among subpopulations defined by different socioeconomic characteristics.

**Design, Setting, and Participants:**

This cohort study used longitudinal data on participants with electronic health records (EHRs) from the All of Us Research Program between calendar years 2016 and 2022. Statistical analysis was performed from September 25, 2023, through February 23, 2024.

**Exposure:**

Satellite-derived 5-year mean PM_2.5_ exposure at the 3-digit zip code level according to participants’ residential address.

**Main Outcome and Measures:**

Incident myocardial infarction (MI) and stroke were obtained from the EHRs. Stratified Cox proportional hazards regression models were used to estimate the hazard ratio (HR) between PM_2.5_ exposure and incident MI or stroke. We evaluated subpopulations defined by 3 socioeconomic characteristics: contextual deprivation (less deprived, more deprived), annual household income (≥$50 000, <$50 000), and race and ethnicity (non-Hispanic Black, non-Hispanic White). We calculated the ratio of HRs (RHR) to quantify disparities between these subpopulations.

**Results:**

A total of 210 554 participants were analyzed (40% age >60 years; 59.4% female; 16.7% Hispanic, 19.4% Non-Hispanic Black, 56.1% Non-Hispanic White, 7.9% other [American Indian, Asian, more than 1 race and ethnicity]), among whom 954 MI and 1407 stroke cases were identified. Higher PM_2.5_ levels were associated with higher MI and stroke risks. However, disadvantaged groups (more deprived, income <$50 000 per year, Black race) were more vulnerable to high PM_2.5_ levels. The disparities were most pronounced between groups defined by contextual deprivation. For instance, increasing PM_2.5_ from 6 to 10 μg/m^3^, the HR for stroke was 1.13 (95% CI, 0.85-1.51) in the less-deprived vs 2.57 (95% CI, 2.06-3.21) in the more-deprived cohort; 1.46 (95% CI, 1.07-2.01) in the $50 000 or more per year vs 2.27 (95% CI, 1.73-2.97) in the under $50 000 per year cohort; and 1.70 (95% CI, 1.35-2.16) in White individuals vs 2.76 (95% CI, 1.89-4.02) in Black individuals. The RHR was highest for contextual deprivation (2.27; 95% CI, 1.59-3.24), compared with income (1.55; 95% CI, 1.05-2.29) and race and ethnicity (1.62; 95% CI, 1.02-2.58).

**Conclusions and Relevance:**

In this cohort study, while individual race and ethnicity and income remained crucial in the adverse association of PM_2.5_ with cardiovascular risks, contextual deprivation was a more robust socioeconomic characteristic modifying the association of PM_2.5_ exposure.

## Key Points

**Question:**

Compared with individual-level factors, do contextual disadvantages modify the risk of fine particulate matter (PM_2.5_) exposure with cardiovascular health?

**Findings:**

In this cohort study, using data on 210 554 individuals from the All of Us Research Program, disparities in associations of PM_2.5_ exposure with myocardial infarction and stroke were most pronounced between subpopulations characterized by contextual deprivation. The disparities were less with individual race and ethnicity or income.

**Meaning:**

While individual race and ethnicity and income remain crucial when considering the adverse association between PM_2.5_ and cardiovascular outcomes, the findings of this study suggest that contextual deprivation is a more robust socioeconomic characteristic modifying the association between cardiovascular health and PM_2.5_.

## Teaser

This cohort study examines the association between air pollution and cardiovascular disease by contextual deprivation in addition to race and ethnicity and income alone.

## Introduction

A large body of evidence supports the association between exposure to air pollution, especially fine particulate matter (PM_2.5_), and cardiovascular disease (CVD).^1,2,3,4^ The PM_2.5_ indicator refers to airborne particles with an aerodynamic diameter of less than 2.5 μm. Due to their small size, these particles can penetrate deep into the respiratory tract, posing substantial health risks. To protect the general population, both the US Environmental Protection Agency and World Health Organization set guidelines for ambient PM_2.5_ exposure.^5,6^ As a result of these efforts, a decreasing trend in the average ambient PM_2.5_ level has been observed across the contiguous US over the past decades, roughly from 15 μg/m^3^ in 2000 to 8 μg/m^3^ in 2016.^7,8,9^ Despite the improvement in air quality, exposure disparities persist, with the socioeconomically disadvantaged subpopulations remaining the most exposed over time.^7,9,10,11^

Within this context, several recent studies have assessed whether health benefits of the decreasing PM_2.5_ level are distributed equitably across different subpopulations in the US.^12,13,14,15^ Race and ethnicity is the most frequently investigated characteristic, as studies consistently observed that the non-Hispanic Black population bore the highest health burdens associated with PM_2.5_ exposure, reflected as a higher PM_2.5_-attributable mortality risk.^12,13^ Research on the role of income also concluded that low-income individuals would benefit more from lower PM_2.5_ levels compared with high-income individuals.^13^

While individual characteristics, such as race and ethnicity and income, have been identified as key factors affecting vulnerability to PM_2.5_, there is a need to prioritize our understanding of contextual deprivation when examining PM_2.5_ exposure and its health implications.^16,17^ Contextual deprivation encompasses the collective challenges faced by a community or population, including, but not limited to, poverty levels, quality of housing, and employment opportunities.^16,17^ In 2008, the US National Institutes of Health proposed a multilevel conceptual model with an emphasis on contextual deprivation as a guideline for future investigation.^18^ A prior study suggested that individual socioeconomic advantages were not sufficient to protect individuals against the adverse influence of contextual deprivation on health.^19^ Focusing on individual characteristics alone can inadvertently overlook the intertwined nature of individuals with their surrounding context,^20,21,22^ thus preventing us from adopting a more holistic perspective to address the root causes of health disparities.

To better assess the PM_2.5_-related health disparities in the US, we analyzed the electronic health record (EHR) data of more than 210 000 participants aged 36 years or older in the All of Us Research Program.^23^ Our analysis examined ambient PM_2.5_ exposure and incident CVD. Specifically, we considered 2 CVD emergencies in this study: myocardial infarction (MI) and stroke, because of their substantial role in CVD mortality. We estimated their nonlinear exposure-response curves in association with PM_2.5_ exposure. We stratified the study population by race and ethnicity, household income, and contextual deprivation, aiming to understand the outcomes associated with individual and contextual socioeconomic characteristics.

## Methods

### The All of Us Research Program

Our study adhered to the Strengthening the Reporting of Observational Studies in Epidemiology (STROBE) reporting guideline for cohort studies. The All of Us Research Program is a prospective cohort that currently includes more than 544 000 adults living in the US and its territories initiated in 2017. The goals, recruitment methods and sites, and scientific rationale for All of Us have been described.^23^ All of Us data include participants’ responses to a series of questionnaires, physical measurements collected by study staff at enrollment, and information from participants’ EHRs. The data are made available to researchers via the Researcher Workbench. The study was approved and overseen by the All of Us institutional review board. Informed consent was waived because of the use of deidentified archival data. This study used the All of Us data at the controlled tier released as of February 15, 2023. The present study used data from calendar years 2016 to 2022, and statistical analysis was performed from September 25, 2023, through February 23, 2024 We restricted the study population to those with valid EHR and residential data. We restricted our analysis to participants aged 36 years or older because of the low risk of MI or stroke among the younger population.^24^

### EHR-Derived Diagnoses

Electronic health record–derived diagnoses were determined using Systematized Nomenclature of Medicine–Clinical Terms codes and mapped to Observational Health and Medicines Outcomes Partnership concept ID by the All of Us Data and Research Center. The full list of concepts used to determine MI and stroke can be found in the eMethods 1 and eMethods 2 in Supplement 1.

We identified primary diagnoses or conditions of MI and stroke after enrollment (ie, incident MI and stroke) from EHRs. For participants with incident MI and/or stroke, the follow-up time was calculated as the difference between enrollment and the initial diagnosis. For participants without an outcome of interest, the follow-up time was calculated as the difference between enrollment and December 31, 2022, or death, whichever came earlier. We also retrieved atherosclerotic CVD history, hypertension status, and type 2 diabetes status from EHRs. Hypertension was defined as 2 or more hypertension diagnoses and/or description and at least 1 hypertension medication prescription in EHRs.^25^

### Measures

All of Us participants answered the Basics and Lifestyle questionnaires when they were enrolled. The Basics questionnaire elicits demographic information including age, race and ethnicity, educational level, marital status, household income, and geography. The Lifestyle questionnaire collects data on the use of tobacco and alcohol.^26^

Based on these survey questionnaires, we retrieved data on age, gender (male, female, and other), self-reported race and ethnicity (Hispanic, non-Hispanic Black [hereinafter, Black], non-Hispanic White [hereinafter, White], and Other [American Indian, Asian, >1 race and ethnicity, and groups in addition to those listed]), household income (<$35 000, $35 000-<$50 000,≥$50 000-$75 000, $75 000-$150 000, and>$150 000 per year), smoking status (never, former, and current), and health insurance status (no or yes). Body mass index was calculated as weight in kilograms divided by height in meters squared, using the height and weight measured at enrollment and grouped into underweight (<18.5), normal weight (18.5-25), overweight (25-30), and obese (>30) categories.

### PM_2.5_ Exposure Assessment

The PM_2.5_ exposure data were obtained from the Atmospheric Composition Analysis Group at Washington University at St Louis. Annual surface PM_2.5_ levels were estimated using a satellite-derived model.^27,28,29^ The resultant values showed great cross-validated agreement (*R*^2^ = 0.99). The datasets at a 0.01° × 0.01° scale (approximately 1.1 km^2^) for surface PM_2.5_ levels were used in this study.

The All of Us data contain the 3-digit residential zip code for each participant as of the 2023 release due to privacy concerns. We therefore averaged PM_2.5_ levels during the 5 years preceding the end of follow-up on all 0.01° × 0.01° grids within the 3-digit zip code to address the temporal variability of PM_2.5_. This approach captures the cumulative exposure effect, offering a comprehensive assessment of long-term exposures and commonly used in prior studies.^1,2,3^ eFigure 1 in Supplement 1 shows the distribution of All of Us participants.

### Contextual Deprivation

Contextual deprivation was represented using the deprivation index in this study. The deprivation index is a composite score based on 6 different socioeconomic variables at the community level, including poverty prevalence, household income, educational level, insurance prevalence, reliance on public assistance, and vacant house prevalence.^30^ The deprivation index was normalized to a range from 0 to 1, with a higher index indicating more deprivation. In this study, we stratified the study population into 2 groups based on the median: less deprived (lower than the median) and more deprived (higher than the median).

### Statistical Analysis

To estimate the nonlinear exposure-response curve, we fit stratified Cox proportional hazards regression models with penalized splines for PM_2.5_ exposure to estimate the pointwise hazard ratios (HRs) and corresponding 95% CIs. The stratified terms included age at enrollment, race and ethnicity, sex, household income, and contextual deprivation. The models were also adjusted for potential confounders, including body mass index, health insurance status, smoking status, hypertension status, atherosclerotic CVD history, type 2 diabetes, penalized splines for the average temperature over the same period,^31^ and average nitrogen dioxide concentration between 2017 and 2019, the latest data for other air pollutant we could find during the study period.^32^ A full description of the model can be found in eMethods 3 in Supplement 1. Missing data were imputed using the random forest imputation algorithm.^33^ We used 6 μg/m^3^ as the reference value and present the results for exposure level from 6 to 12 μg/m^3^.

We ran the regression models in the subpopulations of race and ethnicity (Black vs White), household income (<$50 000 vs≥$50 000 per year), and contextual deprivation (less deprived vs more deprived). For race and ethnicity, we present the comparison between the Black and White cohorts. The exposure-response results for the Hispanic population are available in eFigure 4 in Supplement 1.

To statistically compare the exposure-estimate curves between subpopulations, we calculated the pointwise ratio of HRs (RHR) and corresponding 95% CI for each socioeconomic characteristic (ie, race and ethnicity, household income, and contextual deprivation). The RHR was calculated as the HR in disadvantaged groups divided by the HR in advantaged groups. The 95% CI was generated using bootstrapping.

We conducted several sensitivity analyses to examine the robustness of our results: (1) using exposure 3 years preceding the end of follow-up, (2) excluding patients with a history of MI or stroke, (3) excluding individuals living at the current address for less than 3 years, and (4) specifying the degree of freedom as 3 and placing knots at the tertiles for the penalized splines for PM_2.5_. The statistical analysis was performed using the survival package in R, version 4.4.0 (R Foundation for Statistical Computing).

## Results

### Study Population Characteristics

The study population included 210 554 participants across the US and 690 311 person-years through December 31, 2022 (Table; eFigure 1 in Supplement 1). Among them, 40% were older than 60 years, 59.4% female, 38.4% male, 19.4% Black, 16.7% Hispanic, and 56.1% White. A total of 954 incident MIs (incidence rate, 0.14) and 1407 incident strokes (incidence rate, 0.20) were identified from the EHRs. The incidence rates of MI and stroke were higher in the less-deprived group than the more-deprived group (MI: 0.16 vs 0.12 per 100 person-years; stroke: 0.23 vs 0.18 per 100 person-years). In comparison, the incidence rates were higher in the disadvantaged groups of race and ethnicity and household income.

**Table.** Distributions of Selected Characteristics in the Overall Study Population and Stratified Populations in the All of Us Research Program

Characteristic	No. (%)						
	Total (N = 210 554)	Contextual deprivation		Annual household income, $		Race and ethnicity	
		Less deprived (n = 108 438)	More deprived (n = 102 116)	≥50 000 (n = 84 840)	<50 000 (n = 81 327)	Non-Hispanic Black (n = 40 767)	Non-Hispanic White (n = 118 049)
Incident MI							
Incident No.	954	604	350	309	432	288	521
Incidence rate, per 100 person-years	0.14	0.16	0.12	0.12	0.15	0.20	0.14
Incident stroke							
Incident No.	1407	853	554	426	634	426	732
Incidence rate, per 100 person-years	0.20	0.23	0.18	0.16	0.23	0.30	0.19
Atherosclerotic CVD history							
No	185 918 (88.3)	94 871 (87.5)	91 047 (89.2)	75 672 (89.2)	71 507 (87.9)	36 571 (89.7)	102 395 (86.7)
Yes	24 636 (11.7)	13 567 (12.5)	11 069 (10.8)	9168 (10.8)	9820 (12.1)	4196 (10.3)	15 654 (13.3)
Hypertension							
No	115 250 (54.7)	59 872 (55.2)	55 378 (54.2)	52 132 (61.4)	40 636 (50.0)	18 434 (45.2)	66 632 (56.4)
Yes	95 304 (45.3)	48 566 (44.8)	46 738 (45.8)	32 708 (38.6)	40 691 (50.0)	22 333 (54.8)	51 417 (43.6)
Type 2 diabetes							
No	187 067 (88.8)	96 172 (88.7)	90 895 (89.0)	77 804 (91.7)	70 615 (86.8)	33 849 (83.0)	107 589 (91.1)
Yes	23 487 (11.2)	12 266 (11.3)	11 221 (11.0)	7036 (8.3)	10 712 (13.2)	6918 (17.0)	10 460 (8.9)
Sex							
Female	125 101 (59.4)	64 500 (59.5)	60 601 (59.3)	50 051 (59.0)	49 196 (60.5)	23 802 (58.4)	69 513 (58.9)
Male	80 945 (38.4)	41 646 (38.4)	39 299 (38.5)	34 209 (40.3)	30 896 (38.0)	16 239 (39.8)	47 474 (40.2)
Other	2329 (1.1)	1210 (1.1)	969 (0.9)	530 (0.6)	1091 (1.3)	638 (1.6)	964 (0.8)
Missing	2179 (1.0)	1082 (1.0)	1247 (1.2)	50 (0.1)	144 (0.2)	88 (0.2)	98 (0.1)
Age, y							
36-50	52 391 (24.9)	24 882 (23.0)	27 509 (26.9)	20 338 (24.0)	21 813 (26.8)	10 090 (24.8)	24 011 (20.3)
51-65	73 935 (35.1)	35 250 (32.5)	38 685 (37.9)	26 230 (30.9)	31 210 (38.4)	19 373 (47.5)	35 413 (30.0)
>65	84 228 (40.0)	48 306 (44.5)	35 922 (35.2)	38 272 (45.1)	28 304 (34.8)	11 304 (27.7)	58 625 (49.7)
BMI^a^							
Underweight	2321 (1.1)	1095 (1.0)	1226 (1.2)	731 (0.9)	1015 (1.2)	624 (1.5)	1240 (1.1)
Normal	47 465 (22.5)	25 806 (23.8)	21 659 (21.2)	22 120 (26.1)	15 718 (19.3)	7868 (19.3)	29 160 (24.7)
Overweight	62 941 (29.9)	33 988 (31.3)	28 953 (28.4)	27 872 (32.9)	21 904 (26.9)	10 380 (25.5)	36 658 (31.1)
Obese	89 192 (42.4)	43 318 (39.9)	45 874 (44.9)	30 944 (36.5)	39 850 (49.0)	21 186 (52.0)	45 350 (38.4)
Missing	8635 (4.1)	4231 (3.9)	4404 (4.3)	3173 (3.7)	2840 (3.5)	709 (1.7)	5641 (4.8)
Smoking status							
Never	113 858 (54.1)	60 196 (55.5)	53 662 (52.6)	52 722 (62.1)	36 233 (44.6)	18 853 (46.2)	62 588 (53.0)
Former	56 217 (26.7)	31 684 (29.2)	24 533 (24.0)	26 265 (31.0)	19 744 (24.3)	6693 (16.4)	39 156 (33.2)
Current	34 694 (16.5)	13 763 (12.7)	20 931 (20.5)	4020 (4.7)	23 018 (28.3)	13 644 (33.5)	13 647 (11.6)
Missing	5785 (2.7)	2795 (2.6)	2990 (2.9)	1833 (2.2)	2332 (2.9)	1577 (3.9)	2658 (2.3)
Health insurance							
Uninsured	10 182 (4.8)	2818 (2.6)	7364 (7.2)	633 (0.7)	6048 (7.4)	3718 (9.1)	2534 (2.1)
Insured	193 191 (91.8)	102 549 (94.6)	90 642 (88.8)	83 693 (98.6)	72 998 (89.8)	35 449 (87.0)	114 213 (96.8)
Missing	7181 (3.4)	3071 (2.8)	4110 (4.0)	514 (0.6)	2281 (2.8)	1600 (3.9)	1302 (1.1)
Contextual deprivation	115 594 (54.9)						
Less deprived	94 960 (45.1)	NA	NA	56 231 (66.3)	38 587 (47.4)	12 277 (30.1)	78 214 (66.3)
More deprived	115 594 (54.9)	NA	NA	28 609 (33.7)	42 740 (52.6)	28 490 (69.9)	39 835 (33.7)
Household income, $ per year							
<35 000	65 776 (31.2)	27 481 (25.3)	38 295 (37.5)	NA	NA	22 507 (55.2)	25 649 (21.7)
35 000-<50 000	15 551 (7.4)	8164 (7.5)	7387 (7.2)	NA	NA	2544 (6.2)	9811 (8.3)
50 000-<75 000	21 588 (10.3)	12 150 (11.2)	9438 (9.2)	NA	NA	2434 (6.0)	15 667 (13.3)
≥75 000-<150 000	38 834 (18.4)	24 380 (22.5)	14 454 (14.2)	NA	NA	2274 (5.6)	31 080 (26.3)
≥150 000	24 418 (11.6)	16 973 (15.7)	7445 (7.3)	NA	NA	741 (1.8)	20 305 (17.2)
Missing	44 387 (21.1)	19 290 (17.8)	25 097 (24.6)	NA	NA	10 267 (25.2)	15 537 (13.2)
Race and ethnicity							
Hispanic	35 091 (16.7)	13 951 (12.9)	21 140 (20.7)	6003 (7.1)	15 979 (19.6)	NA	NA
Non-Hispanic Black	40 767 (19.4)	11 742 (10.8)	29 025 (28.4)	5449 (6.4)	25 051 (30.8)	NA	NA
Non-Hispanic White	118 049 (56.1)	73 483 (67.8)	44 566 (43.6)	67 052 (79.0)	35 460 (43.6)	NA	NA
Other^b^	16 647 (7.9)	9262 (8.5)	7385 (7.2)	6336 (7.5)	4837 (5.9)	NA	NA

Abbreviations: BMI, body mass index; CVD, cardiovascular disease; MI, myocardial infarction; NA, not applicable.

^a^ Body mass index (calculated as weight in kilograms divided by height in meters squared): underweight, less than 18.5; normal weight, 18.5 to 25; overweight, 25 to 30; and obese, greater than 30.

^b^ This category includes respondents who identified their race and ethnicity as American Indian, Asian, more than 1 race and ethnicity, and any other of those listed.

**Figure 1.**
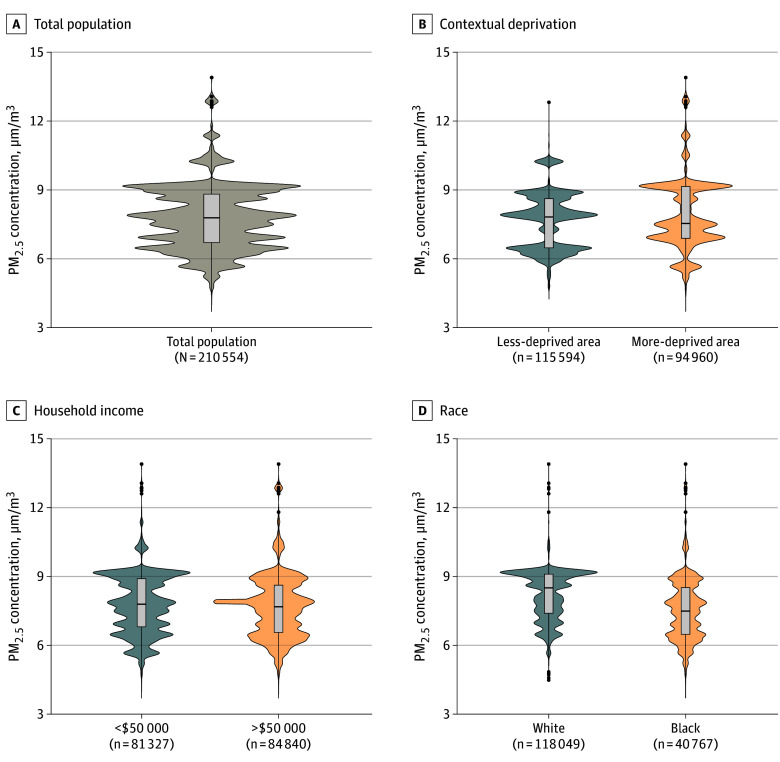
Distributions of 5-Year Mean Fine Particulate Matter (PM_2.5_) Exposure Levels in All of Us Participants

The mean (SD) 5-year PM_2.5_ exposure level during the study period was 7.7 (1.4) μg/m^3^ across the overall study population (Figure 1; eTable 1 in Supplement 1). White participants were exposed to lower PM_2.5_ levels than Black participants (mean [SD], 7.5 [1.3] vs 8.2 [1.1] μg/m^3^). The exposure levels were similar between household income and contextual deprivation groups. Crude Kaplan-Meier analysis showed that the group of both higher PM_2.5_ exposure (higher than the median) and socioeconomic disadvantages had the greatest risk for MI and stroke (eFigure 2 in Supplement 1).

### Risk Associated With PM_2.5_

**Figure 2.**
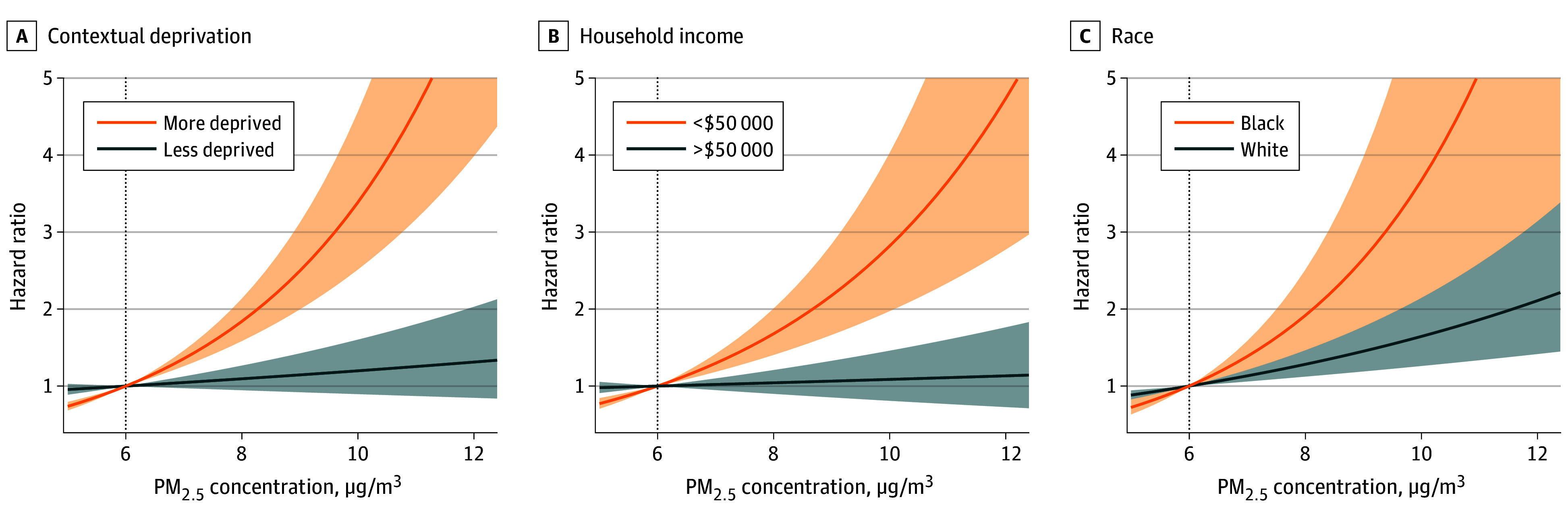
Exposure-Response Curves for the Association Between Fine Particulate Matter (PM_2.5_) Exposure and Incident Myocardial Infarction

**Figure 3.**
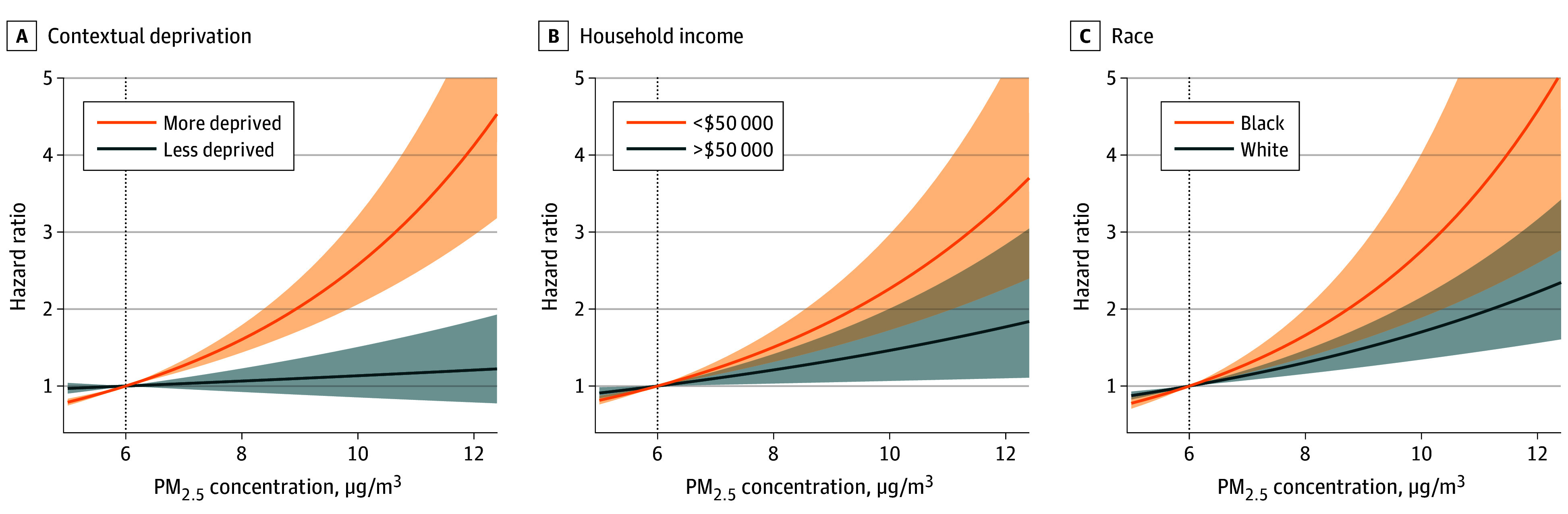
Exposure-Response Curves for the Association Between Fine Particulate Matter (PM_2.5_) Exposure and Incident Stroke

When the PM_2.5_ exposure level increased from 6 to 12 μg/m^3^, we observed increasing risks of incident MI and stroke along the continuum (eFigure 3 in Supplement 1). When stratified by socioeconomic characteristics, we observed differing exposure-response curves between the subpopulations. Overall, disadvantaged groups (ie, more deprived, household income<$50 000 per year, and Black) showed steeper exposure-response curves compared with advantaged groups, suggesting that the disadvantaged groups were more vulnerable to the detrimental association of high PM_2.5_ exposures (Figure 2 and Figure 3; original values are presented in eTable 2 and eTable 3 in Supplement 1).

**Figure 4.**
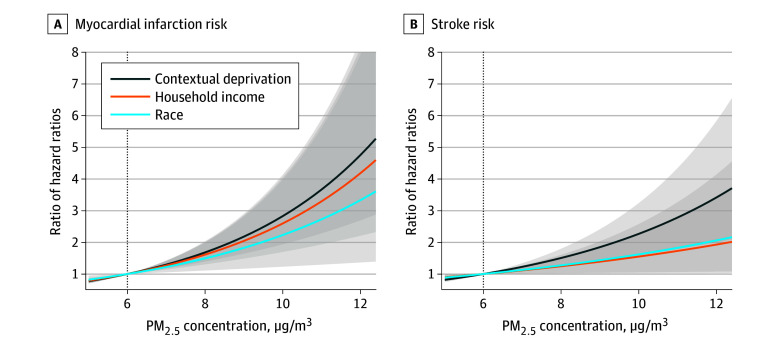
Ratio of Hazard Ratios for Myocardial Infarction or Stroke Between Different Subpopulations

For MI (Figure 2), increasing exposure levels from 6 to 10 μg/m^3^ was associated with an HR of 1.20 (95% CI, 0.90-1.60) in the less-deprived group vs 3.39 (95% CI, 2.51-4.56) in the more-deprived group, corresponding to an RHR of 2.83 (95% CI, 1.93-4.13) (Figure 4; original values are presented in eTable 4 in Supplement 1). When the exposure level was further increased, the RHR for contextual deprivation was strengthened to 4.75 (95% CI, 2.69-8.39) for 12 μg/m^3^. A similar pattern was observed in subpopulations defined by household income and racial and ethnic groups. Specifically, the RHR for 12 μg/m^3^ was 4.18 (95% CI, 2.20-7.92) for household income groups and 3.33 (95% CI, 1.36-8.12) for racial and ethnic groups.

We observed similar increasing risks for stroke that the disparities increased in subpopulations along with PM_2.5_ exposure increase (Figure 3). Briefly, with increasing PM_2.5_ from 6 to 10 μg/m^3^, the HR for stroke was 1.13 (95% CI, 0.85-1.51) in the less-deprived vs 2.57 (95% CI, 2.06-3.21) in the more-deprived cohort; 1.46 (95% CI, 1.07-2.01) in the $50 000 or more per year vs 2.27 (95% CI, 1.73-2.97) in the less than $50 000 per year cohort; and 1.70 (95% CI, 1.35-2.16) in White individuals vs 2.76 (95% CI, 1.89-4.02) in Black individuals. The RHR was highest for contextual deprivation (2.27; 95% CI, 1.59-3.24), compared with income (1.55; 95% CI, 1.05-2.29) and race and ethnicity (1.62; 95% CI, 1.02-2.58). Increasing the exposure level from 6 to 12 μg/m^3^ corresponded to RHRs of 3.42 (95% CI, 2.00-5.83) for contextual deprivation groups, 1.93 (95% CI, 1.07-3.46) for household income groups, and 2.06 (95% CI, 1.02-4.14) for racial and ethnic groups. Results for Hispanic people can be found in eFigure 4 in Supplement 1.

In summary, we observed disparities in risks associated with PM_2.5_ exposure in all subpopulations defined by the 3 socioeconomic characteristics for both MI and stroke. The difference was most evident between subpopulations characterized by contextual deprivation instead of race and ethnicity or household income. All sensitivity analyses corroborated that the difference in MI and stroke risks was most pronounced between subpopulations characterized by contextual deprivation (eFigures 5-9 in Supplement 1).

## Discussion

Findings from this study add evidence that lower PM_2.5_ exposure levels would benefit all US residents, as well as across subpopulations defined by different socioeconomic characteristics. Moreover, the results align with prior studies^12,13,14,15^ concluding that socioeconomically disadvantaged groups are more vulnerable to high PM_2.5_ exposure in the US. The present study further illustrates that contextual deprivation, rather than individual race and ethnicity or household income, shows the strongest potential for modifying the association of PM_2.5_ exposure, as disparities in CVD risks are most pronounced between subpopulations defined by contextual deprivation. Our findings suggest a paradigm shift may be warranted, one that pivots from an emphasis on individual-level factors to the wider lens of contextual deprivation. This shift would involve designing policies that not only regulate emissions and reduce ambient pollution levels, but also enhance community resilience and access to clean environments, particularly in underprivileged areas, rather than a focus on individuals.

Based on studies from different disciplines over decades, it has been widely accepted that social structural factors, rather than innate biologic differences, are the primary factor for greater susceptibility to PM_2.5_ exposure.^13,34^ As structural racism stands out as a major cause of health disparities in the US,^35,36^ a large research effort has been devoted to dissecting the nuances of how individual race and ethnicity is associated with environmental health disparities. This focus on individual factors, while yielding critical insights into disparities, may inadvertently overshadow the broader and potentially more impactful role of contextual deprivation. While individual race and ethnicity and income are undeniably crucial factors, to unearth and tackle inequalities more effectively, it is essential to consider the broader concept of contextual deprivation, which indicates the state of disadvantage that arises from the broader social and environmental conditions in which individuals live.^37^ But we should also be reminded that contextual deprivation and individual characteristics are closely interlinked, creating a feedback loop where race and ethnicity can influence the level of contextual deprivation a person experiences, which in turn can affect their health outcomes. This association highlights the need for public health interventions to tackle the broader systemic issues more comprehensively.^38^

### Strengths and Limitations

This study is the one of the first environmental studies from the All of Us Research Program. The present study reflects the latest changes and exposures, providing a more current snapshot of the association between PM_2.5_ exposure and CVD risks and how socioeconomic factors modify the association. Moreover, our research was strengthened by the rich longitudinal nature of the All of Us Research Program, capturing a diverse, population-wide spectrum of participants with ongoing EHRs. This allows a more detailed examination of incident CVD. Another strength of this study is that we are able to include adult participants of all ages. Previous studies have often been constrained by the reliance on ecologic data^12^ or datasets limited to populations aged 65 years or older.^13^ Such constraints potentially skew the understanding of PM_2.5_ impact to reflect predominantly the health outcomes of older adults or render studies prone to ecologic fallacy.

This study has limitations. First, the incidence rates of MI and stroke were 0.14 and 0.20 per 100 person-years in this study. In comparison, the incidence rate reported by the US Centers for Disease Control and Prevention was roughly 0.24 per 100 person-years for both MI and stroke in the general population.^39,40^ However, underdiagnosis appears to be nondifferential across the subpopulations defined by different socioeconomic characteristics. For example, studies reported that the risk of stroke was approximately 1.5 times as high for non-Hispanic Black people as for non-Hispanic White people,^41,42^ while our data also reported a 1.5 times risk of stroke for Black compared with White individuals. Because the present study focuses on the disparities between subpopulations, it is reasonable to conclude that the nondifferential overestimate of MI and stroke should have minimal influence on the observed disparities. Second, the All of Us Research Program has not been linked to the National Death Index yet. Mortality status in All of Us is currently reported by each health provider organization. Therefore, it is possible that some deaths, either from CVD or other causes, were not recorded. However, loss of this information should be nondifferential, because missing information on mortality is not a result of PM_2.5_ exposure. The nondifferential factor would bias the estimates toward the null. Third, the PM_2.5_ exposure was assigned at the 3-digit zip code level, which may not precisely reflect individuals’ exposure levels and thus cause bias. However, using a less fine spatial resolution typically leads to an underestimation of the true effect.^43^ In practice, findings from epidemiologic studies are relatively robust against the exposure error.^43^ Therefore, the exposure at the 3-digit zip code level in this study, although not optimal, still provided crucial evidence for the disparities between subpopulations. Fourth, data on some crucial covariates, including blood cholesterol level, medication prescriptions, physical activity, and other air pollutants during the study period, were not available or only available for a subset of the participants in the recent release. The shorter follow-up in All of Us may also underestimate the risk for MI and stroke in this study. However, our sensitivity analyses suggested that the results are robust in different scenarios.

## Conclusions

While individual race and ethnicity and income remain important socioeconomic factors modifying the association of PM_2.5_ with CVD risks, the findings of this cohort study advocate for a broader approach that emphasizes the influence of contextual deprivation. Future research should incorporate contextual deprivation to develop a more comprehensive understanding of disparities in environmental exposure and health.

## Notes

Supplement 1. **eMethods 1.** Full Observational Health and Medicines Outcomes Partnership (OMOP) Concept List for Heart Attack **eMethods 2.** Full Observational Health and Medicines Outcomes Partnership (OMOP) Concept List for Stroke **eMethods 3.** Full Description of the Statistical Model **eTable 1**. Descriptive Statistics of PM_2.5_ Exposure Level in the Study Population **eTable 2**. Hazard Ratios and 95% Confidence Interval for Heart Attack in Relation to PM_2.5_ Exposure Level With 6 mg/m^3^ as the Reference Level **eTable 3**. Hazard Ratios and 95% Confidence Interval for Stroke in Relation to PM_2.5_ Exposure Level With 6 μg/m^3^ as the Reference Level **eTable 4**. Ratio and Hazard Ratios and 95% Confidence Interval for Heart Attack and Stroke in Relation to PM_2.5_ Exposure Level With 6 μg/m^3^ as the Reference Level Between Different Subpopulations **eFigure 1**. Geographic Distribution of the Study Population **eFigure 2**. Kaplan-Meier Curves for Heart Attack and Stroke According to PM_2.5_ Exposure and Different Socioeconomic Factors **eFigure 3**. Exposure-Response Curves for PM_2.5_ Exposure and Incident Heart Attack or Stroke Risks in the Overall Population **eFigure 4**. Exposure-Response Curves for PM_2.5_ Exposure and Incident Heart Attack or Stroke Risks for Hispanic Population **eFigure 5**. Ratio of Hazard Ratios for Heart Attack or Stroke Between Different Subpopulation Using Exposure 3 Years Preceding the End of Follow-Up **eFigure 6**. Ratio of Hazard Ratios for Heart Attack or Stroke Between Different Subpopulation When Excluding Patients With History of Heart Attack or Stroke **eFigure 7**. Ratio of Hazard Ratios for Heart Attack or Stroke Between Different Subpopulation When Excluding Participants Living at the Current Address for Less Than 3 Years **eFigure 8**. Ratio of Hazard Ratios for Heart Attack or Stroke Between Different Subpopulation When Specifying the Degree of Freedom as 3 for the Penalized Splines **eFigure 9**. Ratio of Hazard Ratios for Heart Attack or Stroke Between Different Subpopulation When Placing the Knots at the Tertiles for the Penalized Splines

Supplement 2.
Data Sharing Statement
