# Supplementary material for: Contextual Deprivation, Race and Ethnicity, and Income in Air Pollution and Cardiovascular Disease
Source: JAMA Netw Open. 2024 Aug 19;7(8):e2429137. doi: 10.1001/jamanetworkopen.2024.29137 (PMC11333981; doi:10.1001/jamanetworkopen.2024.29137)
Supplement: Supplement 1. — eMethods 1. Full Observational Health and Medicines Outcomes Partnership (OMOP) Concept List for Heart Attack eMethods 2. Full Observational Health and Medicines Outcomes Partnership (OMOP) Concept List for Stroke eMethods 3. Full Description of the Statistical Model eTable 1. Descriptive Statistics of PM2.5 Exposure Level in the Study Population eTable 2. Hazard Ratios and 95% Confidence Interval for Heart Attack in Relation to PM2.5 Exposure Level With 6 mg/m3 as the Reference Level eTable 3. Hazard Ratios and 95% Confidence Interval for Stroke in Relation to PM2.5 Exposure Level With 6 μg/m3 as the Reference Level eTable 4. Ratio and Hazard Ratios and 95% Confidence Interval for Heart Attack and Stroke in Relation to PM2.5 Exposure Level With 6 μg/m3 as the Reference Level Between Different Subpopulations eFigure 1. Geographic Distribution of the Study Population eFigure 2. Kaplan-Meier Curves for Heart Attack and Stroke According to PM2.5 Exposure and Different Socioeconomic Factors eFigure 3. Exposure-Response Curves for PM2.5 Exposure and Incident Heart Attack or Stroke Risks in the Overall Population eFigure 4. Exposure-Response Curves for PM2.5 Exposure and Incident Heart Attack or Stroke Risks for Hispanic Population eFigure 5. Ratio of Hazard Ratios for Heart Attack or Stroke Between Different Subpopulation Using Exposure 3 Years Preceding the End of Follow-Up eFigure 6. Ratio of Hazard Ratios for Heart Attack or Stroke Between Different Subpopulation When Excluding Patients With History of Heart Attack or Stroke eFigure 7. Ratio of Hazard Ratios for Heart Attack or Stroke Between Different Subpopulation When Excluding Participants Living at the Current Address for Less Than 3 Years eFigure 8. Ratio of Hazard Ratios for Heart Attack or Stroke Between Different Subpopulation When Specifying the Degree of Freedom as 3 for the Penalized Splines eFigure 9. Ratio of Hazard Ratios for Heart Attack or Stroke Between Different Subpopulation When Placing the Knots [file jamanetwopen-e2429137-s001.pdf]

Supplementary Online Content

Luo J, Craver A, Jin Z, et al. Contextual deprivation, race and ethnicity, and income in the association between air pollution and cardiovascular disease. *JAMA Netw Open*. 2024;7(8):e2429137. doi:10.1001/jamanetworkopen.2024.29137

eMethods 1. Full Observational Health and Medicines Outcomes Partnership (OMOP) Concept List for Heart Attack

eMethods 2. Full Observational Health and Medicines Outcomes Partnership (OMOP) Concept List for Stroke

eMethods 3. Full Description of the Statistical Model

eTable 1. Descriptive Statistics of $PM_{2.5}$ Exposure Level in the Study Population

eTable 2. Hazard Ratios and 95% Confidence Interval for Heart Attack in Relation to $PM_{2.5}$ Exposure Level With 6 mg/m^3 as the Reference Level

eTable 3. Hazard Ratios and 95% Confidence Interval for Stroke in Relation to $PM_{2.5}$ Exposure Level With 6 μg/m^3 as the Reference Level

eTable 4. Ratio and Hazard Ratios and 95% Confidence Interval for Heart Attack and Stroke in Relation to $PM_{2.5}$ Exposure Level With 6 μg/m^3 as the Reference Level Between Different Subpopulations

eFigure 1. Geographic Distribution of the Study Population

eFigure 2. Kaplan-Meier Curves for Heart Attack and Stroke According to PM2.5 Exposure and Different Socioeconomic Factors

eFigure 3. Exposure-Response Curves for PM2.5 Exposure and Incident Heart Attack or Stroke Risks in the Overall Population

eFigure 4. Exposure-Response Curves for PM2.5 Exposure and Incident Heart Attack or Stroke Risks for Hispanic Population

eFigure 5. Ratio of Hazard Ratios for Heart Attack or Stroke Between Different Subpopulation Using Exposure 3 Years Preceding the End of Follow-Up

eFigure 6. Ratio of Hazard Ratios for Heart Attack or Stroke Between Different Subpopulation When Excluding Patients With History of Heart Attack or Stroke

eFigure 7. Ratio of Hazard Ratios for Heart Attack or Stroke Between Different Subpopulation When Excluding Participants Living at the Current Address for Less Than 3 Years

eFigure 8. Ratio of Hazard Ratios for Heart Attack or Stroke Between Different Subpopulation When Specifying the Degree of Freedom as 3 for the Penalized Splines

eFigure 9. Ratio of Hazard Ratios for Heart Attack or Stroke Between Different Subpopulation When Placing the Knots at the Tertiles for the Penalized Splines

This supplementary material has been provided by the authors to give readers additional information about their work.

© 2024 Luo J et al. *JAMA Network Open.*

eMethods 1: Full Observational Health and Medicines Outcomes Partnership (OMOP) concept list for heart attack

1) myocardial infarction, 2) old myocardial infarction, 3) old anterior myocardial infarction, 4) old inferior myocardial infarction, 5) acute myocardial infarction, 6) acute non-st segment elevation myocardial infarction, 7) acute st segment elevation myocardial infarction, 8) acute st segment elevation myocardial infarction involving left anterior descending coronary artery, 9) acute st segment elevation myocardial infarction due to right coronary artery occlusion, 10) acute st segment elevation myocardial infarction due to left coronary artery occlusion, 11) acute st segment elevation myocardial infarction, 12) acute subendocardial infarction, 13) acute myocardial infarction of anterior wall, 14) acute anterior st segment elevation myocardial infarction, 15) acute myocardial infarction of inferior wall, 16) acute myocardial infarction due to right coronary artery occlusion, 17) acute st segment elevation myocardial infarction due to right coronary artery occlusion, 18) acute myocardial infarction of anterolateral wall, 19) acute myocardial infarction of inferoposterior wall, 20) acute myocardial infarction of inferolateral wall, 21) acute myocardial infarction of lateral wall, 22) acute myocardial infarction due to left coronary artery occlusion, 23) acute st segment elevation myocardial infarction due to left coronary artery occlusion, 24) acute myocardial infarction of apical-lateral wall, 25) acute myocardial infarction of septum, 26) acute non-q wave infarction, 27) acute non-q wave infarction - widespread, 28) acute widespread myocardial infarction, 29) subendocardial myocardial infarction, 30) acute subendocardial infarction, 31) myocardial infarction due to demand ischemia, 32) subsequent myocardial infarction, 33) subsequent non-st segment elevation myocardial infarction, 34) subsequent st segment elevation myocardial infarction, 35) subsequent myocardial infarction of anterior wall, 36) subsequent myocardial infarction of

© 2024 Luo J et al. *JAMA Network Open.*

inferior wall, 37) true posterior myocardial infarction, 38) myocardial infarction in recovery phase, 39) non-q wave myocardial infarction, 40) heart attack

© 2024 Luo J et al. *JAMA Network Open.*

eMethods 2: Full Observational Health and Medicines Outcomes Partnership (OMOP) concept list for stroke

1) thrombotic stroke, 2) cerebellar stroke syndrome, 3) cerebrovascular accident due to thrombus of basilar artery, 4) cerebrovascular accident due to thrombus of left carotid artery, 5) cerebrovascular accident due to thrombus of left middle cerebral artery, 6) cerebrovascular accident due to thrombus of right carotid artery, 7) cerebrovascular accident due to thrombus of right cerebellar artery, 8) cerebrovascular accident due to thrombus of right middle cerebral artery, 9) cerebrovascular accident due to thrombus of right posterior cerebral artery, 10) left posterior cerebral artery thrombosis with stroke, 11) embolic stroke, 12) cardioembolic stroke, 13) brainstem stroke syndrome, 14) posterior inferior cerebellar artery syndrome, 15) ischemic stroke, 16) occlusive stroke, 17) multi-infarct dementia, 18) multi-infarct dementia, uncomplicated, 19) multi-infarct dementia with delirium, 20) multi-infarct dementia with delusions, 21) multi-infarct dementia with depression, 22) occlusion of cerebral artery with stroke, 23) acute stroke, 24) progressing stroke, 25) basilar artery embolism with stroke, 26) cerebrovascular accident due to cerebral artery occlusion, 27) cerebrovascular accident due to occlusion of left posterior cerebral artery, 28) left posterior cerebral artery thrombosis with stroke, 29) left anterior cerebral artery embolism with stroke, 30) cerebellar stroke, 31) cerebral infarction due to stenosis of carotid artery, 32) cerebrovascular accident due to occlusion of left anterior cerebral artery, 33) cerebrovascular accident due to occlusion of left carotid artery, 34) left carotid artery embolism with stroke, 35) cerebrovascular accident due to occlusion of left vertebral artery, 36) left vertebral artery embolism with stroke, 37) cerebrovascular accident due to right carotid artery occlusion, 38) right carotid artery embolism with stroke, 39) cerebrovascular accident due to right carotid artery stenosis, 40) cerebrovascular accident due to

right vertebral artery occlusion, 41) right vertebral artery embolism with stroke, 42) cerebrovascular accident due to stenosis of left vertebral artery, 43) cerebrovascular accident due to thrombus of right vertebral artery, 44) cerebrovascular accident with intracranial hemorrhage, 45) chronic cerebrovascular accident, 46) cerebral infarction, 47) cerebral infarction due to thrombosis of cerebral arteries, 48) cerebral infarction due to thrombosis of middle cerebral artery, 49) cerebral infarction due to embolism of cerebral arteries, 50) infarction - precerebral, 51) cerebral infarct due to thrombosis of precerebral arteries, 52) cerebral infarction due to embolism of precerebral arteries, 53) cerebral infarction due to embolism of middle cerebral artery, 54) cerebral infarction due to occlusion of precerebral artery, 55) cerebral infarction due to occlusion of basilar artery, 56) cerebral infarction due to carotid artery occlusion, 57) cerebral infarction due to vertebral artery occlusion, 58) infarct of cerebrum due to iatrogenic cerebrovascular accident, 59) lacunar infarction, 60) acute lacunar infarction, 61) lacunar ataxic hemiparesis, 62) multiple lacunar infarcts, 63) pure motor lacunar infarction, 64) pure sensory lacunar infarction, 65) cerebral infarction due to cerebral venous thrombosis, 66) cerebral infarction due to cerebral venous thrombosis, non-pyogenic, 67) anterior cerebral circulation infarction, 68) anterior choroidal artery syndrome, 69) cerebral infarction due to cerebral artery occlusion, 70) infarction of basal ganglia, 71) hemorrhagic cerebral infarction, 72) mixed cortical and subcortical vascular dementia, 73) occipital cerebral infarction, 74) pituitary apoplexy, 75) right sided cerebral infarction, 76) thalamic infarction.

© 2024 Luo J et al. *JAMA Network Open.*

eMethods 3: Full description of the statistical model

We opted not to specify pre-set parameters for the spline. Instead, we left the function to automatically determine the optimal parameters. Overall, the model can be expressed as:

$$h(t|X) = h_0(t|age, sex, race, household\ income, contextual\ deprivation)\exp\big(\beta_1(BMI) + \beta_2(health\ insurance\ status) + \beta_3(hypertension) + \beta_4(ASCVD) + \beta_5(smoking\ status) + \beta_6(type\ 2\ diabetes) + \gamma_1 s_1(PM_{2.5}) + \gamma_2 s_2(NO_2) + \gamma_3 s_3(Temperature)\big)$$

where $s(*)$ indicates penalized splines. To adjust for residual autocorrelation within the geographic unit (i.e., 3-digit zip code in this study), we used the generalized estimating equation (GEE) to calculate a statistically robust CI for the effect estimates.

© 2024 Luo J et al. *JAMA Network Open.*

eTable 5. Descriptive statistics of $PM_{2.5}$ exposure level in the study population

Subpopulations	$PM_{2.5}$ exposure level (µg/m^3)						
	Min	Q1	Median	Mean	Q3	Max	SD
All	3.7	6.6	7.7	7.7	8.6	13.9	1.4
Less deprived	4.2	6.5	7.8	7.6	8.5	13.1	1.2
More deprived	3.7	6.9	7.5	7.9	9.1	13.9	1.5
Household income above $50K	3.7	6.5	7.7	7.7	8.6	13.9	1.4
Household income under $50K	3.7	6.8	7.8	7.8	8.9	13.9	1.3
Non-Hispanic White	3.7	6.5	7.5	7.6	8.5	13.9	1.3
Non-Hispanic Black	4.5	7.4	8.5	8.2	9.1	13.9	1.1

© 2024 Luo J et al. *JAMA Network Open.*

eTable 6. Hazard ratios and 95% confidence interval for heart attack in relation to $PM_{2.5}$ exposure level with 6 μg/m^3 as the reference level.

$PM_{2.5}$ exposure level	All	Contextual deprivation		Household income		Race	
		Less deprived	More deprived	Above $50K	Under $50K	Non-Hispanic White	Non-Hispanic Black
5	0.85 (0.81-0.89)	0.96 (0.89-1.03)	0.74 (0.68-0.79)	0.98 (0.91-1.05)	0.77 (0.71-0.84)	0.88 (0.83-0.94)	0.72 (0.63-0.83)
5.2	0.88 (0.85-0.91)	0.96 (0.91-1.02)	0.78 (0.74-0.83)	0.98 (0.93-1.04)	0.81 (0.76-0.87)	0.91 (0.86-0.95)	0.77 (0.69-0.86)
5.4	0.91 (0.88-0.93)	0.97 (0.93-1.02)	0.83 (0.80-0.87)	0.99 (0.94-1.03)	0.86 (0.81-0.90)	0.93 (0.89-0.97)	0.82 (0.76-0.89)
5.6	0.94 (0.92-0.96)	0.98 (0.95-1.01)	0.89 (0.86-0.91)	0.99 (0.96-1.02)	0.90 (0.87-0.93)	0.95 (0.93-0.98)	0.88 (0.83-0.93)
5.8	0.97 (0.96-0.98)	0.99 (0.98-1.01)	0.94 (0.93-0.95)	1.00 (0.98-1.01)	0.95 (0.93-0.97)	0.98 (0.96-0.99)	0.94 (0.91-0.96)
6	1.00 (1.00-1.00)	1.00 (1.00-1.00)	1.00 (1.00-1.00)	1.00 (1.00-1.00)	1.00 (1.00-1.00)	1.00 (1.00-1.00)	1.00 (1.00-1.00)
6.2	1.03 (1.02-1.04)	1.01 (0.99-1.02)	1.06 (1.05-1.08)	1.00 (0.99-1.02)	1.05 (1.03-1.07)	1.03 (1.01-1.04)	1.07 (1.04-1.10)
6.4	1.07 (1.05-1.09)	1.02 (0.99-1.05)	1.13 (1.10-1.16)	1.01 (0.98-1.04)	1.11 (1.07-1.15)	1.05 (1.02-1.08)	1.14 (1.08-1.20)
6.6	1.10 (1.07-1.13)	1.03 (0.98-1.07)	1.20 (1.15-1.26)	1.01 (0.97-1.06)	1.17 (1.11-1.23)	1.08 (1.04-1.12)	1.22 (1.12-1.32)
6.8	1.14 (1.10-1.18)	1.04 (0.98-1.10)	1.28 (1.20-1.35)	1.02 (0.96-1.08)	1.23 (1.15-1.32)	1.10 (1.05-1.16)	1.30 (1.16-1.44)
7	1.18 (1.12-1.23)	1.05 (0.97-1.13)	1.36 (1.26-1.46)	1.02 (0.95-1.10)	1.30 (1.19-1.42)	1.13 (1.06-1.21)	1.38 (1.21-1.58)
7.2	1.21 (1.15-1.29)	1.06 (0.97-1.15)	1.44 (1.32-1.58)	1.03 (0.94-1.12)	1.36 (1.23-1.52)	1.16 (1.07-1.26)	1.48 (1.26-1.73)
7.4	1.25 (1.17-1.34)	1.07 (0.96-1.18)	1.53 (1.38-1.70)	1.03 (0.93-1.14)	1.44 (1.27-1.63)	1.19 (1.08-1.31)	1.58 (1.31-1.90)
7.6	1.30 (1.20-1.40)	1.08 (0.96-1.21)	1.63 (1.45-1.84)	1.03 (0.92-1.16)	1.51 (1.31-1.75)	1.22 (1.10-1.36)	1.68 (1.36-2.08)
7.8	1.34 (1.23-1.46)	1.08 (0.95-1.24)	1.73 (1.51-1.98)	1.04 (0.91-1.19)	1.59 (1.36-1.87)	1.25 (1.11-1.41)	1.79 (1.41-2.28)
8	1.38 (1.26-1.52)	1.09 (0.95-1.27)	1.84 (1.59-2.14)	1.04 (0.90-1.21)	1.68 (1.40-2.01)	1.28 (1.12-1.46)	1.91 (1.46-2.50)
8.2	1.43 (1.29-1.58)	1.10 (0.94-1.30)	1.96 (1.66-2.30)	1.05 (0.89-1.23)	1.77 (1.45-2.15)	1.31 (1.14-1.52)	2.04 (1.52-2.74)
8.4	1.48 (1.32-1.65)	1.11 (0.94-1.33)	2.08 (1.74-2.49)	1.05 (0.88-1.25)	1.86 (1.50-2.31)	1.35 (1.15-1.58)	2.18 (1.58-3.01)
8.6	1.52 (1.35-1.72)	1.12 (0.93-1.36)	2.21 (1.82-2.68)	1.06 (0.87-1.28)	1.96 (1.56-2.47)	1.38 (1.16-1.64)	2.33 (1.64-3.30)
8.8	1.57 (1.38-1.80)	1.14 (0.93-1.39)	2.35 (1.91-2.89)	1.06 (0.86-1.30)	2.07 (1.61-2.65)	1.42 (1.18-1.70)	2.48 (1.70-3.61)
9	1.63 (1.41-1.87)	1.15 (0.92-1.42)	2.50 (2.00-3.12)	1.06 (0.85-1.33)	2.18 (1.67-2.84)	1.45 (1.19-1.77)	2.65 (1.77-3.96)
9.2	1.68 (1.44-1.95)	1.16 (0.92-1.46)	2.65 (2.09-3.37)	1.07 (0.84-1.35)	2.29 (1.72-3.05)	1.49 (1.20-1.84)	2.83 (1.84-4.34)
9.4	1.73 (1.48-2.04)	1.17 (0.91-1.49)	2.82 (2.19-3.63)	1.07 (0.84-1.38)	2.41 (1.78-3.27)	1.53 (1.22-1.91)	3.02 (1.91-4.76)
9.6	1.79 (1.51-2.12)	1.18 (0.91-1.53)	3.00 (2.29-3.92)	1.08 (0.83-1.41)	2.54 (1.84-3.50)	1.56 (1.23-1.99)	3.22 (1.98-5.22)
9.8	1.85 (1.55-2.21)	1.19 (0.90-1.57)	3.19 (2.40-4.23)	1.08 (0.82-1.43)	2.68 (1.91-3.76)	1.60 (1.25-2.06)	3.43 (2.06-5.72)
10	1.91 (1.58-2.31)	1.20 (0.90-1.60)	3.39 (2.51-4.56)	1.09 (0.81-1.46)	2.82 (1.97-4.03)	1.64 (1.26-2.14)	3.66 (2.14-6.27)
10.2	1.98 (1.62-2.41)	1.21 (0.89-1.64)	3.60 (2.63-4.92)	1.09 (0.80-1.49)	2.97 (2.04-4.32)	1.69 (1.28-2.23)	3.91 (2.23-6.87)

© 2024 Luo J et al. *JAMA Network Open.*

10.4	2.04 (1.66-2.51)	1.22 (0.89-1.68)	3.83 (2.76-5.31)	1.10 (0.79-1.52)	3.13 (2.11-4.63)	1.73 (1.29-2.31)	4.17 (2.31-7.53)
10.6	2.11 (1.70-2.62)	1.23 (0.88-1.72)	4.07 (2.89-5.73)	1.10 (0.78-1.54)	3.29 (2.19-4.96)	1.77 (1.31-2.40)	4.45 (2.40-8.25)
10.8	2.18 (1.74-2.73)	1.24 (0.88-1.76)	4.32 (3.02-6.18)	1.10 (0.78-1.57)	3.47 (2.26-5.32)	1.82 (1.32-2.50)	4.75 (2.49-9.05)
11	2.25 (1.78-2.85)	1.25 (0.87-1.80)	4.59 (3.17-6.67)	1.11 (0.77-1.60)	3.65 (2.34-5.70)	1.86 (1.34-2.59)	5.07 (2.59-9.92)
11.2	2.32 (1.82-2.97)	1.27 (0.87-1.85)	4.88 (3.32-7.19)	1.11 (0.76-1.63)	3.85 (2.42-6.12)	1.91 (1.35-2.69)	5.41 (2.69-10.87)
11.4	2.40 (1.86-3.10)	1.28 (0.86-1.89)	5.19 (3.47-7.76)	1.12 (0.75-1.67)	4.05 (2.50-6.56)	1.96 (1.37-2.80)	5.77 (2.80-11.92)
11.6	2.48 (1.90-3.23)	1.29 (0.86-1.94)	5.52 (3.64-8.37)	1.12 (0.74-1.70)	4.27 (2.59-7.03)	2.01 (1.38-2.91)	6.16 (2.91-13.06)
11.8	2.56 (1.95-3.37)	1.30 (0.85-1.98)	5.86 (3.81-9.03)	1.13 (0.74-1.73)	4.49 (2.68-7.54)	2.06 (1.40-3.02)	6.57 (3.02-14.32)
12	2.64 (1.99-3.51)	1.31 (0.85-2.03)	6.23 (3.99-9.74)	1.13 (0.73-1.76)	4.73 (2.77-8.08)	2.11 (1.42-3.14)	7.01 (3.14-15.69)
12.2	2.73 (2.04-3.66)	1.32 (0.84-2.08)	6.62 (4.18-10.51)	1.14 (0.72-1.80)	4.99 (2.87-8.66)	2.16 (1.43-3.26)	7.48 (3.26-17.20)
12.4	2.82 (2.09-3.82)	1.34 (0.84-2.13)	7.04 (4.37-11.34)	1.14 (0.71-1.83)	5.25 (2.97-9.29)	2.22 (1.45-3.39)	7.99 (3.38-18.85)
12.6	2.91 (2.13-3.98)	1.35 (0.83-2.18)	7.48 (4.58-12.23)	1.15 (0.71-1.87)	5.53 (3.07-9.96)	2.27 (1.47-3.52)	8.52 (3.51-20.67)
12.8	3.01 (2.18-4.15)	1.36 (0.83-2.23)	7.95 (4.79-13.20)	1.15 (0.70-1.90)	5.82 (3.18-10.68)	2.33 (1.48-3.65)	9.09 (3.65-22.65)
13	3.11 (2.23-4.33)	1.37 (0.83-2.28)	8.45 (5.02-14.24)	1.16 (0.69-1.94)	6.13 (3.29-11.45)	2.39 (1.50-3.80)	9.70 (3.79-24.83)

© 2024 Luo J et al. *JAMA Network Open.*

eTable 7. Hazard ratios and 95% confidence interval for stroke in relation to $PM_{2.5}$ exposure level with 6 $\mu g/m^3$ as the reference level.

$PM_{2.5}$ exposure level	All	Contextual deprivation		Household income		Race	
		Less deprived	More deprived	Above $50K	Under $50K	Non-Hispanic White	Non-Hispanic Black
5	0.88 (0.84-0.92)	0.97 (0.90-1.04)	0.79 (0.75-0.83)	0.91 (0.84-0.98)	0.82 (0.76-0.87)	0.88 (0.83-0.93)	0.78 (0.71-0.85)
5.2	0.90 (0.87-0.93)	0.98 (0.92-1.03)	0.83 (0.79-0.87)	0.93 (0.87-0.99)	0.85 (0.80-0.90)	0.90 (0.86-0.94)	0.82 (0.76-0.88)
5.4	0.92 (0.90-0.95)	0.98 (0.94-1.02)	0.87 (0.84-0.90)	0.94 (0.90-0.99)	0.88 (0.85-0.92)	0.92 (0.89-0.96)	0.86 (0.81-0.91)
5.6	0.95 (0.93-0.97)	0.99 (0.96-1.02)	0.91 (0.89-0.93)	0.96 (0.93-0.99)	0.92 (0.90-0.95)	0.95 (0.93-0.97)	0.90 (0.87-0.94)
5.8	0.97 (0.97-0.98)	0.99 (0.98-1.01)	0.95 (0.94-0.96)	0.98 (0.97-1.00)	0.96 (0.95-0.97)	0.97 (0.96-0.99)	0.95 (0.93-0.97)
6	1.00 (1.00-1.00)	1.00 (1.00-1.00)	1.00 (1.00-1.00)	1.00 (1.00-1.00)	1.00 (1.00-1.00)	1.00 (1.00-1.00)	1.00 (1.00-1.00)
6.2	1.03 (1.02-1.04)	1.01 (0.99-1.02)	1.05 (1.04-1.06)	1.02 (1.00-1.04)	1.04 (1.03-1.06)	1.03 (1.01-1.04)	1.05 (1.03-1.07)
6.4	1.05 (1.03-1.07)	1.01 (0.98-1.04)	1.10 (1.08-1.12)	1.04 (1.01-1.07)	1.09 (1.06-1.12)	1.05 (1.03-1.08)	1.11 (1.07-1.15)
6.6	1.08 (1.05-1.11)	1.02 (0.98-1.06)	1.15 (1.11-1.19)	1.06 (1.01-1.11)	1.13 (1.09-1.18)	1.08 (1.05-1.12)	1.16 (1.10-1.23)
6.8	1.11 (1.07-1.15)	1.03 (0.97-1.09)	1.21 (1.16-1.26)	1.08 (1.01-1.15)	1.18 (1.12-1.24)	1.11 (1.06-1.17)	1.22 (1.14-1.32)
7	1.14 (1.09-1.19)	1.03 (0.96-1.11)	1.27 (1.20-1.34)	1.10 (1.02-1.19)	1.23 (1.15-1.31)	1.14 (1.08-1.21)	1.29 (1.17-1.42)
7.2	1.17 (1.11-1.23)	1.04 (0.95-1.13)	1.33 (1.24-1.42)	1.12 (1.02-1.23)	1.28 (1.18-1.39)	1.17 (1.09-1.26)	1.36 (1.21-1.52)
7.4	1.20 (1.13-1.28)	1.04 (0.95-1.15)	1.39 (1.29-1.50)	1.14 (1.02-1.28)	1.33 (1.21-1.46)	1.20 (1.11-1.31)	1.43 (1.25-1.63)
7.6	1.23 (1.15-1.32)	1.05 (0.94-1.18)	1.46 (1.34-1.59)	1.16 (1.03-1.32)	1.39 (1.24-1.55)	1.24 (1.13-1.36)	1.50 (1.29-1.75)
7.8	1.26 (1.17-1.37)	1.06 (0.93-1.20)	1.53 (1.38-1.69)	1.19 (1.03-1.37)	1.44 (1.28-1.63)	1.27 (1.14-1.41)	1.58 (1.33-1.87)
8	1.30 (1.19-1.42)	1.06 (0.92-1.23)	1.60 (1.44-1.79)	1.21 (1.03-1.42)	1.51 (1.31-1.72)	1.31 (1.16-1.47)	1.66 (1.37-2.01)
8.2	1.33 (1.21-1.47)	1.07 (0.92-1.25)	1.68 (1.49-1.90)	1.23 (1.04-1.47)	1.57 (1.35-1.82)	1.34 (1.18-1.53)	1.75 (1.42-2.15)
8.4	1.37 (1.23-1.52)	1.08 (0.91-1.28)	1.76 (1.54-2.01)	1.26 (1.04-1.52)	1.63 (1.39-1.92)	1.38 (1.19-1.59)	1.84 (1.47-2.31)
8.6	1.40 (1.25-1.58)	1.09 (0.90-1.31)	1.85 (1.60-2.13)	1.28 (1.04-1.57)	1.70 (1.43-2.03)	1.41 (1.21-1.65)	1.93 (1.51-2.47)
8.8	1.44 (1.27-1.63)	1.09 (0.89-1.33)	1.94 (1.66-2.26)	1.31 (1.05-1.63)	1.77 (1.47-2.14)	1.45 (1.23-1.71)	2.03 (1.56-2.65)
9	1.48 (1.29-1.69)	1.10 (0.89-1.36)	2.03 (1.72-2.40)	1.33 (1.05-1.69)	1.85 (1.51-2.26)	1.49 (1.25-1.78)	2.14 (1.61-2.84)
9.2	1.52 (1.31-1.75)	1.11 (0.88-1.39)	2.13 (1.78-2.54)	1.36 (1.05-1.75)	1.92 (1.55-2.39)	1.53 (1.27-1.85)	2.25 (1.66-3.05)
9.4	1.56 (1.34-1.81)	1.11 (0.87-1.42)	2.23 (1.85-2.69)	1.38 (1.06-1.81)	2.00 (1.59-2.52)	1.57 (1.29-1.92)	2.37 (1.72-3.26)
9.6	1.60 (1.36-1.88)	1.12 (0.87-1.45)	2.34 (1.92-2.85)	1.41 (1.06-1.87)	2.09 (1.64-2.67)	1.62 (1.31-2.00)	2.49 (1.77-3.50)
9.8	1.64 (1.38-1.94)	1.13 (0.86-1.48)	2.45 (1.99-3.03)	1.44 (1.06-1.94)	2.17 (1.68-2.81)	1.66 (1.33-2.08)	2.62 (1.83-3.75)
10	1.68 (1.41-2.01)	1.13 (0.85-1.51)	2.57 (2.06-3.21)	1.46 (1.07-2.01)	2.27 (1.73-2.97)	1.70 (1.35-2.16)	2.76 (1.89-4.02)
10.2	1.73 (1.43-2.08)	1.14 (0.85-1.54)	2.70 (2.14-3.40)	1.49 (1.07-2.08)	2.36 (1.78-3.14)	1.75 (1.37-2.24)	2.90 (1.95-4.31)

© 2024 Luo J et al. *JAMA Network Open.*

10.4	1.77 (1.46-2.16)	1.15 (0.84-1.57)	2.83 (2.22-3.60)	1.52 (1.07-2.15)	2.46 (1.82-3.31)	1.80 (1.39-2.33)	3.05 (2.01-4.62)
10.6	1.82 (1.48-2.24)	1.16 (0.83-1.60)	2.96 (2.30-3.82)	1.55 (1.08-2.23)	2.56 (1.87-3.50)	1.85 (1.41-2.42)	3.21 (2.08-4.96)
10.8	1.87 (1.51-2.31)	1.16 (0.83-1.64)	3.11 (2.38-4.05)	1.58 (1.08-2.31)	2.67 (1.93-3.70)	1.89 (1.43-2.52)	3.38 (2.15-5.31)
11	1.92 (1.53-2.40)	1.17 (0.82-1.67)	3.26 (2.47-4.29)	1.61 (1.08-2.39)	2.78 (1.98-3.90)	1.95 (1.45-2.61)	3.55 (2.22-5.70)
11.2	1.97 (1.56-2.48)	1.18 (0.81-1.70)	3.41 (2.56-4.55)	1.64 (1.09-2.47)	2.90 (2.03-4.12)	2.00 (1.47-2.72)	3.74 (2.29-6.11)
11.4	2.02 (1.59-2.57)	1.18 (0.81-1.74)	3.58 (2.66-4.82)	1.67 (1.09-2.56)	3.02 (2.09-4.35)	2.05 (1.49-2.82)	3.93 (2.36-6.55)
11.6	2.07 (1.61-2.66)	1.19 (0.80-1.78)	3.75 (2.75-5.11)	1.70 (1.09-2.65)	3.14 (2.15-4.59)	2.11 (1.51-2.93)	4.14 (2.44-7.02)
11.8	2.13 (1.64-2.76)	1.20 (0.79-1.81)	3.93 (2.85-5.42)	1.74 (1.10-2.74)	3.27 (2.21-4.85)	2.16 (1.54-3.05)	4.35 (2.52-7.53)
12	2.18 (1.67-2.86)	1.21 (0.79-1.85)	4.12 (2.96-5.74)	1.77 (1.10-2.84)	3.41 (2.27-5.12)	2.22 (1.56-3.17)	4.58 (2.60-8.07)
12.2	2.24 (1.70-2.96)	1.21 (0.78-1.89)	4.32 (3.07-6.09)	1.80 (1.11-2.94)	3.55 (2.33-5.41)	2.28 (1.58-3.29)	4.82 (2.68-8.65)
12.4	2.30 (1.73-3.06)	1.22 (0.78-1.93)	4.53 (3.18-6.45)	1.84 (1.11-3.05)	3.70 (2.40-5.71)	2.34 (1.61-3.42)	5.07 (2.77-9.27)
12.6	2.36 (1.76-3.17)	1.23 (0.77-1.97)	4.75 (3.30-6.84)	1.87 (1.11-3.15)	3.86 (2.46-6.03)	2.41 (1.63-3.56)	5.33 (2.86-9.94)
12.8	2.42 (1.79-3.28)	1.24 (0.76-2.01)	4.98 (3.42-7.25)	1.91 (1.12-3.27)	4.02 (2.53-6.37)	2.47 (1.66-3.69)	5.61 (2.95-10.66)
13	2.49 (1.82-3.40)	1.25 (0.76-2.05)	5.22 (3.55-7.69)	1.95 (1.12-3.38)	4.18 (2.60-6.73)	2.54 (1.68-3.84)	5.90 (3.05-11.43)

© 2024 Luo J et al. *JAMA Network Open.*

eTable 8. Ratio and hazard ratios and 95% confidence interval for heart attack and stroke in relation to $PM_{2.5}$ exposure level with 6 μg/m^3 as the reference level between different subpopulations

$PM_{2.5}$ exposure level	Heart attack			Stroke		
	Contextual deprivation	Household income	Race	Contextual deprivation	Household income	Race
5	0.77 (0.70-0.85)	0.79 (0.71-0.88)	0.82 (0.71-0.95)	0.81 (0.75-0.89)	0.90 (0.81-0.99)	0.89 (0.79-1.00)
5.2	0.81 (0.75-0.88)	0.83 (0.76-0.90)	0.85 (0.76-0.96)	0.85 (0.79-0.91)	0.92 (0.85-0.99)	0.91 (0.83-1.00)
5.4	0.86 (0.81-0.91)	0.87 (0.81-0.92)	0.89 (0.81-0.97)	0.88 (0.84-0.93)	0.94 (0.88-0.99)	0.93 (0.87-1.00)
5.6	0.90 (0.87-0.94)	0.91 (0.87-0.95)	0.92 (0.87-0.98)	0.92 (0.89-0.95)	0.96 (0.92-1.00)	0.95 (0.91-1.00)
5.8	0.95 (0.93-0.97)	0.95 (0.93-0.97)	0.96 (0.93-0.99)	0.96 (0.94-0.98)	0.98 (0.96-1.00)	0.98 (0.95-1.00)
6	1.00 (1.00-1.00)	1.00 (1.00-1.00)	1.00 (1.00-1.00)	1.00 (1.00-1.00)	1.00 (1.00-1.00)	1.00 (1.00-1.00)
6.2	1.05 (1.03-1.07)	1.05 (1.03-1.07)	1.04 (1.01-1.07)	1.04 (1.02-1.06)	1.02 (1.00-1.04)	1.02 (1.00-1.05)
6.4	1.11 (1.07-1.15)	1.10 (1.05-1.15)	1.08 (1.02-1.15)	1.09 (1.05-1.12)	1.04 (1.00-1.09)	1.05 (1.00-1.10)
6.6	1.17 (1.10-1.24)	1.15 (1.08-1.23)	1.13 (1.03-1.23)	1.13 (1.07-1.19)	1.07 (1.01-1.13)	1.07 (1.00-1.15)
6.8	1.23 (1.14-1.33)	1.21 (1.11-1.32)	1.17 (1.04-1.32)	1.18 (1.10-1.26)	1.09 (1.01-1.18)	1.10 (1.00-1.21)
7	1.30 (1.18-1.43)	1.27 (1.14-1.41)	1.22 (1.05-1.42)	1.23 (1.12-1.34)	1.12 (1.01-1.23)	1.13 (1.00-1.27)
7.2	1.37 (1.22-1.53)	1.33 (1.17-1.51)	1.27 (1.06-1.52)	1.28 (1.15-1.42)	1.14 (1.01-1.28)	1.16 (1.00-1.33)
7.4	1.44 (1.26-1.64)	1.40 (1.20-1.62)	1.32 (1.07-1.63)	1.33 (1.18-1.51)	1.17 (1.02-1.34)	1.18 (1.01-1.39)
7.6	1.52 (1.30-1.76)	1.46 (1.23-1.74)	1.38 (1.09-1.75)	1.39 (1.20-1.60)	1.19 (1.02-1.39)	1.21 (1.01-1.46)
7.8	1.60 (1.35-1.89)	1.54 (1.27-1.86)	1.43 (1.10-1.87)	1.45 (1.23-1.70)	1.22 (1.02-1.45)	1.24 (1.01-1.53)
8	1.68 (1.39-2.03)	1.61 (1.30-1.99)	1.49 (1.11-2.01)	1.51 (1.26-1.80)	1.24 (1.02-1.51)	1.27 (1.01-1.61)
8.2	1.77 (1.44-2.18)	1.69 (1.34-2.14)	1.55 (1.12-2.16)	1.57 (1.29-1.91)	1.27 (1.03-1.58)	1.30 (1.01-1.68)
8.4	1.87 (1.49-2.34)	1.77 (1.37-2.29)	1.62 (1.13-2.31)	1.63 (1.32-2.02)	1.30 (1.03-1.64)	1.34 (1.01-1.77)
8.6	1.96 (1.54-2.51)	1.86 (1.41-2.45)	1.68 (1.14-2.48)	1.70 (1.35-2.15)	1.33 (1.03-1.71)	1.37 (1.01-1.85)
8.8	2.07 (1.59-2.70)	1.95 (1.45-2.63)	1.75 (1.16-2.66)	1.77 (1.38-2.28)	1.36 (1.03-1.78)	1.40 (1.01-1.94)
9	2.18 (1.64-2.90)	2.04 (1.48-2.81)	1.82 (1.17-2.85)	1.85 (1.41-2.41)	1.39 (1.04-1.86)	1.44 (1.01-2.04)
9.2	2.30 (1.69-3.11)	2.14 (1.52-3.02)	1.90 (1.18-3.06)	1.93 (1.45-2.56)	1.42 (1.04-1.94)	1.47 (1.01-2.13)
9.4	2.42 (1.75-3.34)	2.25 (1.56-3.23)	1.98 (1.19-3.28)	2.01 (1.48-2.71)	1.45 (1.04-2.02)	1.51 (1.01-2.24)
9.6	2.55 (1.81-3.58)	2.36 (1.61-3.46)	2.06 (1.20-3.51)	2.09 (1.52-2.88)	1.48 (1.04-2.10)	1.54 (1.01-2.35)
9.8	2.68 (1.87-3.85)	2.47 (1.65-3.71)	2.14 (1.22-3.77)	2.18 (1.55-3.05)	1.52 (1.05-2.19)	1.58 (1.02-2.46)
10	2.83 (1.93-4.13)	2.59 (1.69-3.97)	2.23 (1.23-4.04)	2.27 (1.59-3.24)	1.55 (1.05-2.29)	1.62 (1.02-2.58)
10.2	2.98 (2.00-4.43)	2.72 (1.74-4.26)	2.32 (1.24-4.33)	2.36 (1.63-3.43)	1.58 (1.05-2.38)	1.66 (1.02-2.70)

© 2024 Luo J et al. *JAMA Network Open.*

10.4	3.14 (2.07-4.76)	2.85 (1.78-4.56)	2.42 (1.26-4.65)	2.46 (1.66-3.64)	1.62 (1.05-2.48)	1.70 (1.02-2.84)
10.6	3.30 (2.14-5.11)	2.99 (1.83-4.89)	2.51 (1.27-4.98)	2.56 (1.70-3.86)	1.65 (1.06-2.59)	1.74 (1.02-2.97)
10.8	3.48 (2.21-5.48)	3.14 (1.88-5.24)	2.62 (1.28-5.34)	2.67 (1.74-4.10)	1.69 (1.06-2.70)	1.78 (1.02-3.12)
11	3.66 (2.28-5.89)	3.29 (1.93-5.61)	2.72 (1.29-5.73)	2.78 (1.78-4.34)	1.73 (1.06-2.81)	1.83 (1.02-3.27)
11.2	3.86 (2.36-6.32)	3.45 (1.98-6.01)	2.84 (1.31-6.14)	2.90 (1.82-4.61)	1.77 (1.06-2.93)	1.87 (1.02-3.43)
11.4	4.07 (2.44-6.78)	3.62 (2.04-6.44)	2.95 (1.32-6.59)	3.02 (1.87-4.89)	1.81 (1.07-3.05)	1.92 (1.02-3.59)
11.6	4.28 (2.52-7.28)	3.80 (2.09-6.90)	3.07 (1.34-7.07)	3.15 (1.91-5.18)	1.85 (1.07-3.18)	1.96 (1.02-3.77)
11.8	4.51 (2.60-7.82)	3.98 (2.15-7.40)	3.20 (1.35-7.58)	3.28 (1.96-5.49)	1.89 (1.07-3.32)	2.01 (1.02-3.95)
12	4.75 (2.69-8.39)	4.18 (2.20-7.92)	3.33 (1.36-8.12)	3.42 (2.00-5.83)	1.93 (1.07-3.46)	2.06 (1.02-4.14)
12.2	5.00 (2.78-9.01)	4.38 (2.26-8.49)	3.46 (1.38-8.71)	3.56 (2.05-6.18)	1.97 (1.08-3.60)	2.11 (1.02-4.34)
12.4	5.27 (2.87-9.67)	4.60 (2.32-9.10)	3.61 (1.39-9.34)	3.71 (2.10-6.55)	2.01 (1.08-3.76)	2.16 (1.03-4.55)
12.6	5.55 (2.97-10.38)	4.82 (2.38-9.75)	3.75 (1.41-10.02)	3.86 (2.15-6.95)	2.06 (1.08-3.91)	2.21 (1.03-4.77)
12.8	5.85 (3.07-11.14)	5.06 (2.45-10.44)	3.91 (1.42-10.74)	4.02 (2.20-7.37)	2.10 (1.09-4.08)	2.27 (1.03-5.01)
13	6.16 (3.17-11.96)	5.30 (2.51-11.19)	4.07 (1.44-11.52)	4.19 (2.25-7.82)	2.15 (1.09-4.25)	2.32 (1.03-5.25)

© 2024 Luo J et al. *JAMA Network Open.*

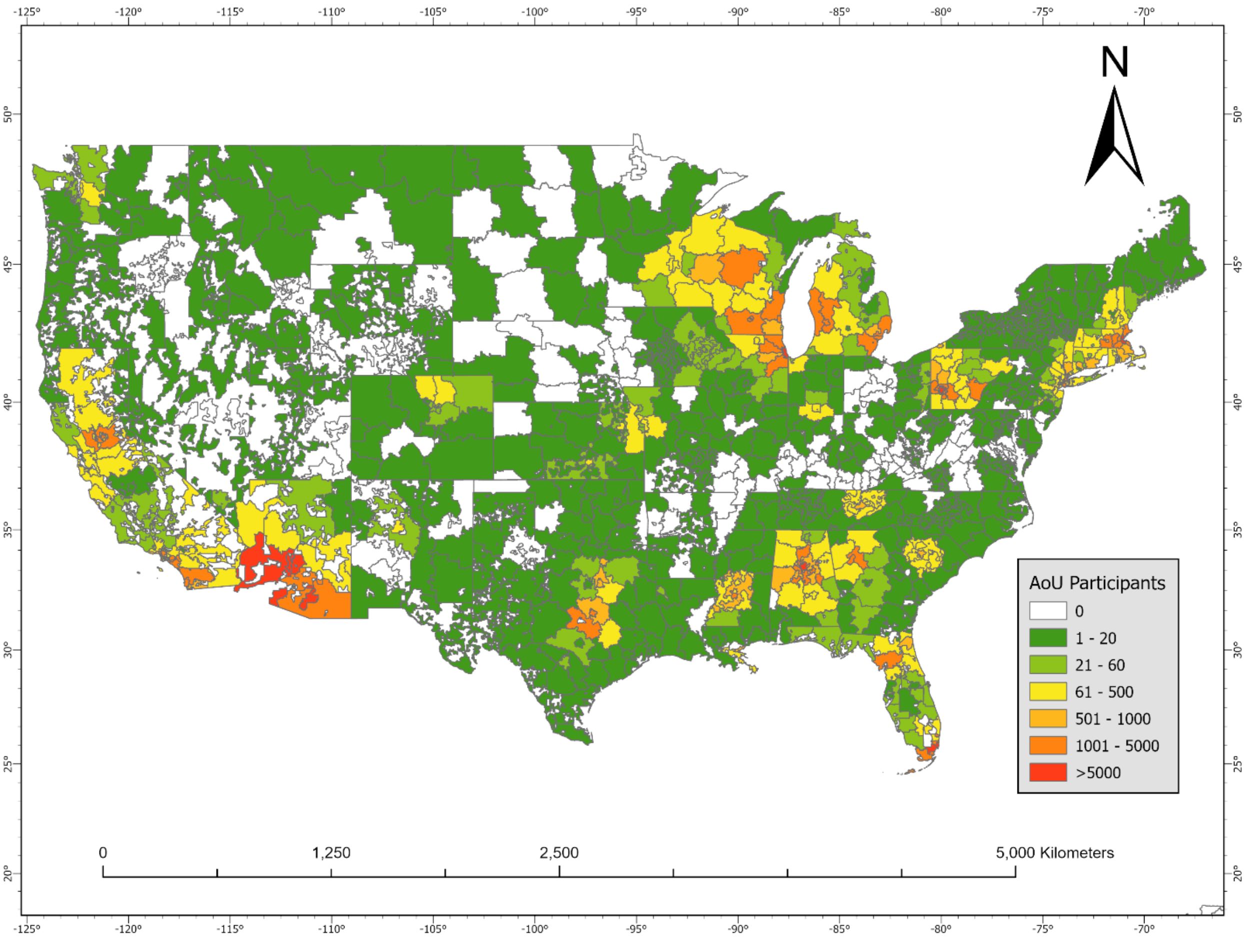

eFigure 10. Geographic distribution of the study population

© 2024 Luo J et al. *JAMA Network Open.*

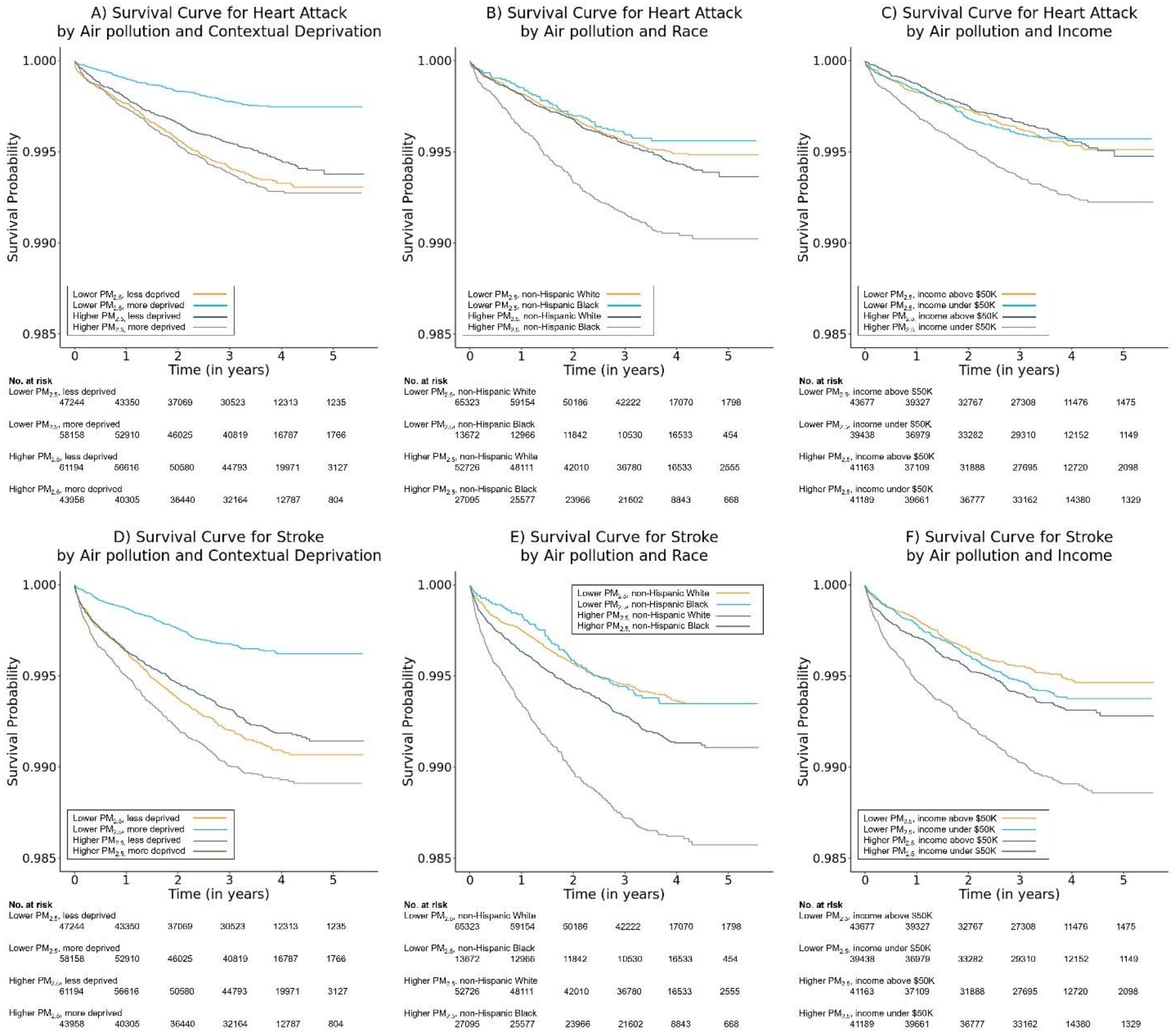

eFigure 11. Kaplan-Meier curves for heart attack and stroke according to PM2.5 exposure and different socioeconomic factors. A) Risk of heart attack by contextual deprivation. B) Risk of heart attack by race. C) Risk of heart attack by household income. D) Risk of stroke by contextual deprivation. E) Risk of stroke by race. F) Risk of stroke by household income

© 2024 Luo J et al. *JAMA Network Open.*

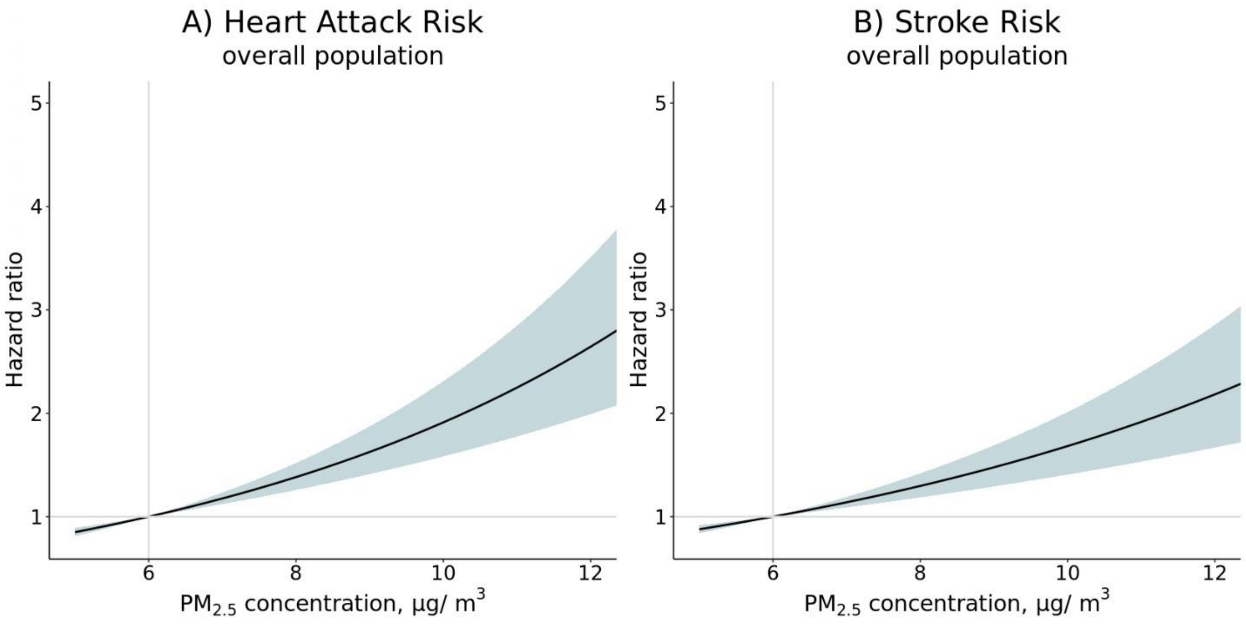

eFigure 12. Exposure-response curves for PM2.5 exposure and incident heart attack or stroke risks in the overall population

© 2024 Luo J et al. *JAMA Network Open.*

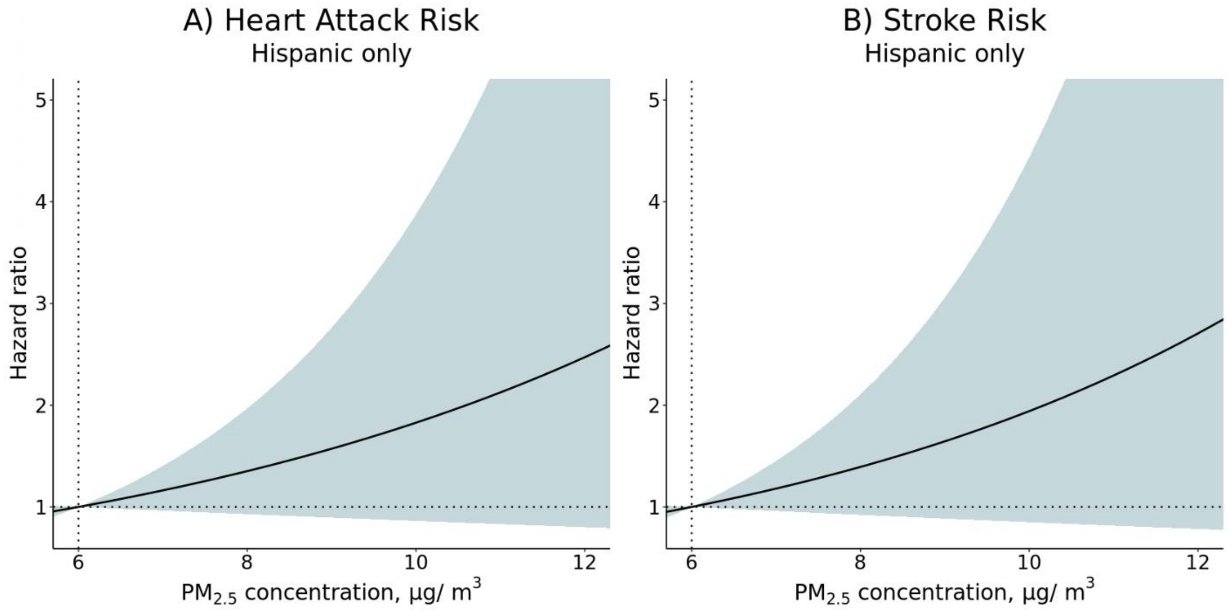

eFigure 13. Exposure-response curves for PM2.5 exposure and incident heart attack or stroke risks for Hispanic population

© 2024 Luo J et al. *JAMA Network Open.*

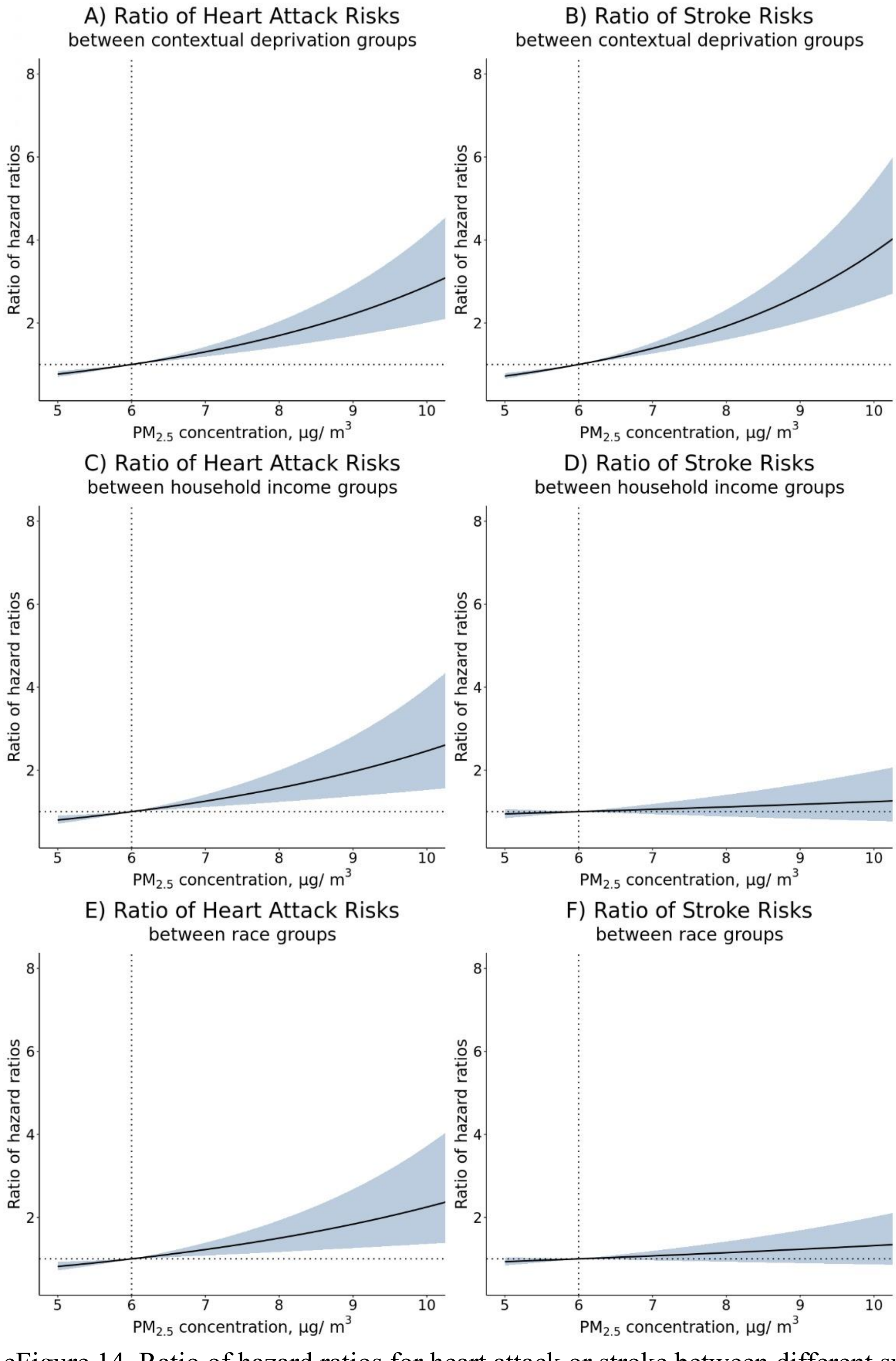

eFigure 14. Ratio of hazard ratios for heart attack or stroke between different subpopulation using exposure 3 years preceding the end of follow-up.

© 2024 Luo J et al. *JAMA Network Open.*

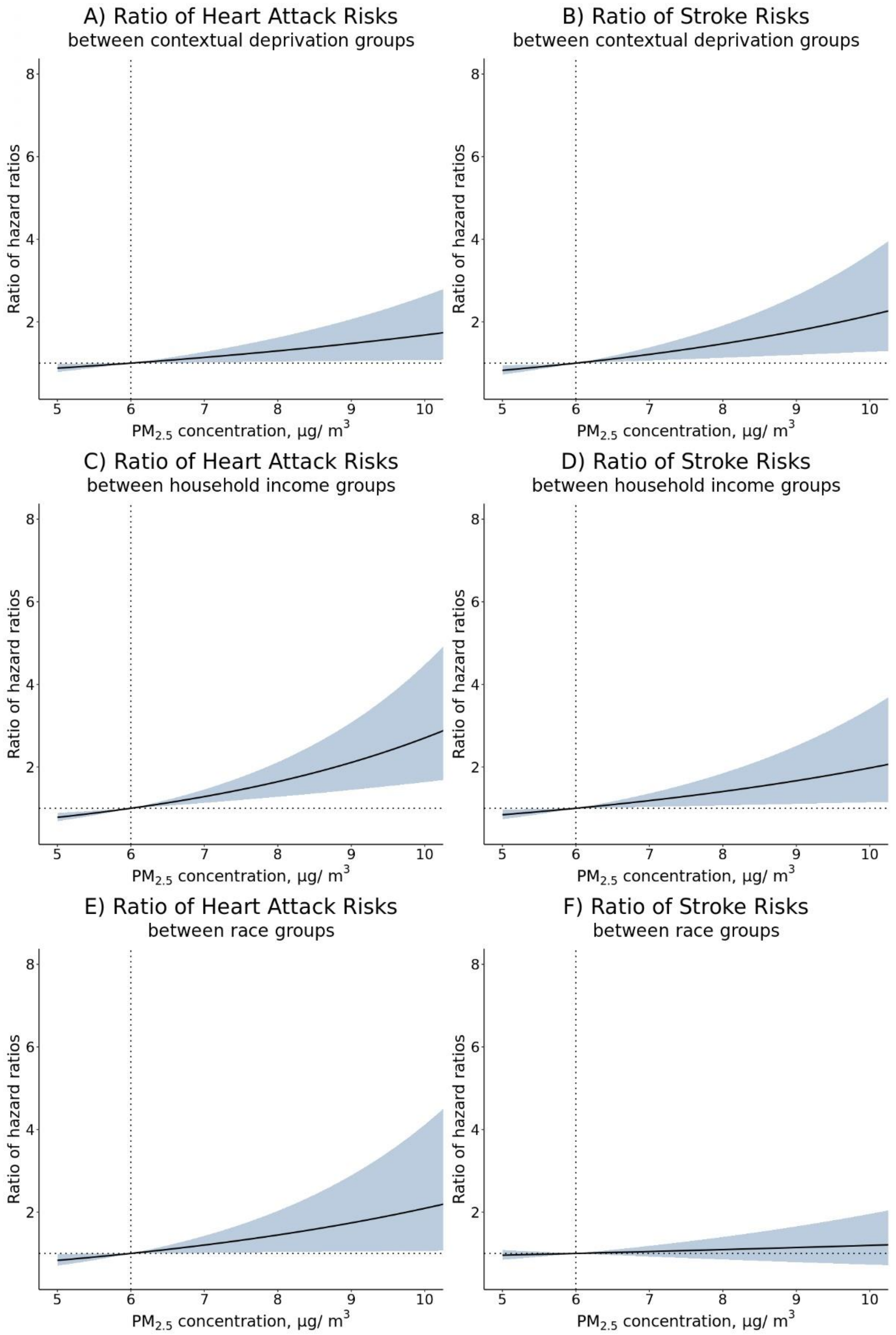

eFigure 15. Ratio of hazard ratios for heart attack or stroke between different subpopulation when excluding patients with history of heart attack or stroke.

© 2024 Luo J et al. *JAMA Network Open.*

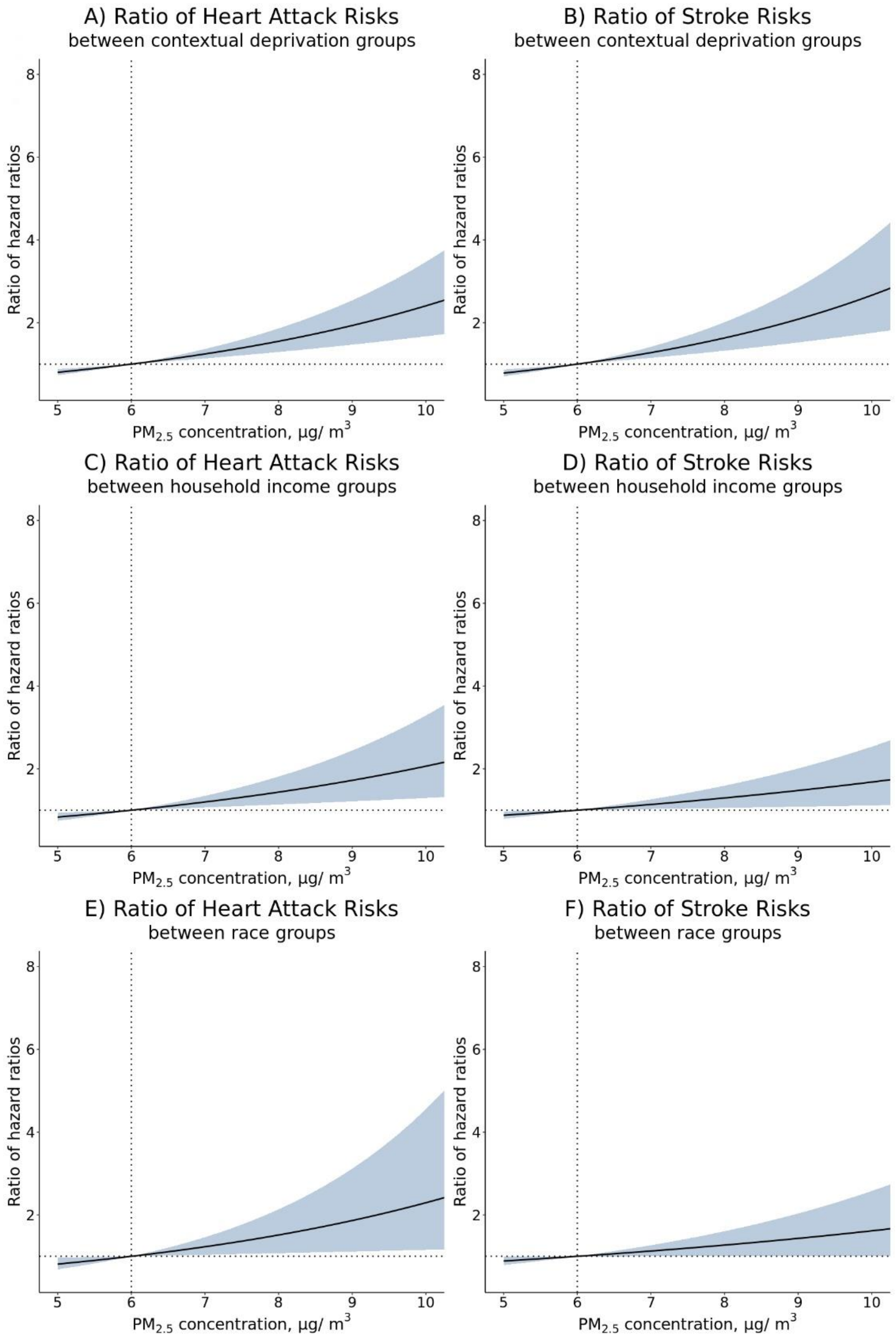

eFigure 16. Ratio of hazard ratios for heart attack or stroke between different subpopulation when excluding participants living at the current address for less than 3 years.

© 2024 Luo J et al. *JAMA Network Open.*

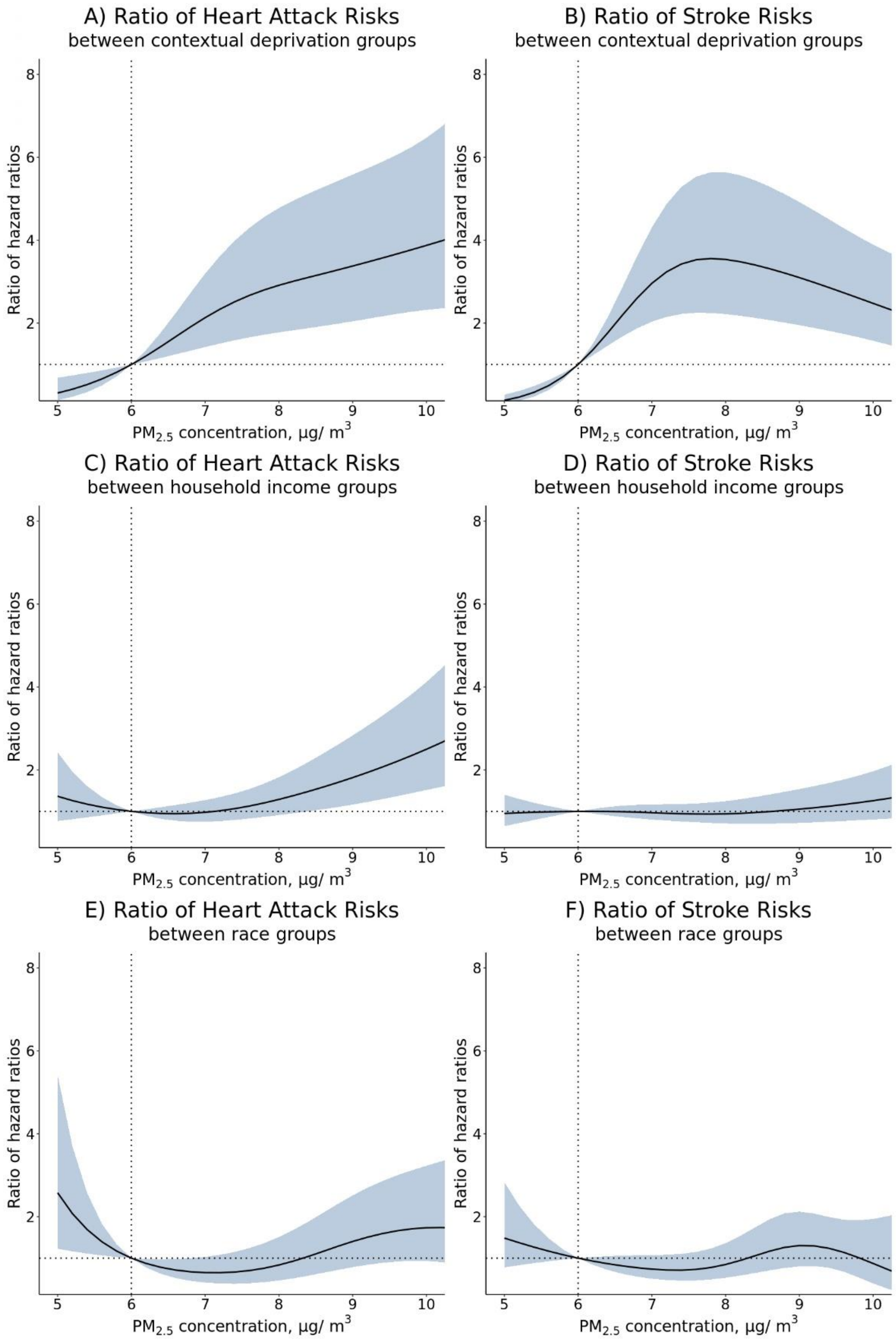

eFigure 17. Ratio of hazard ratios for heart attack or stroke between different subpopulation when specifying the degree of freedom as 3 for the penalized splines.

© 2024 Luo J et al. *JAMA Network Open.*

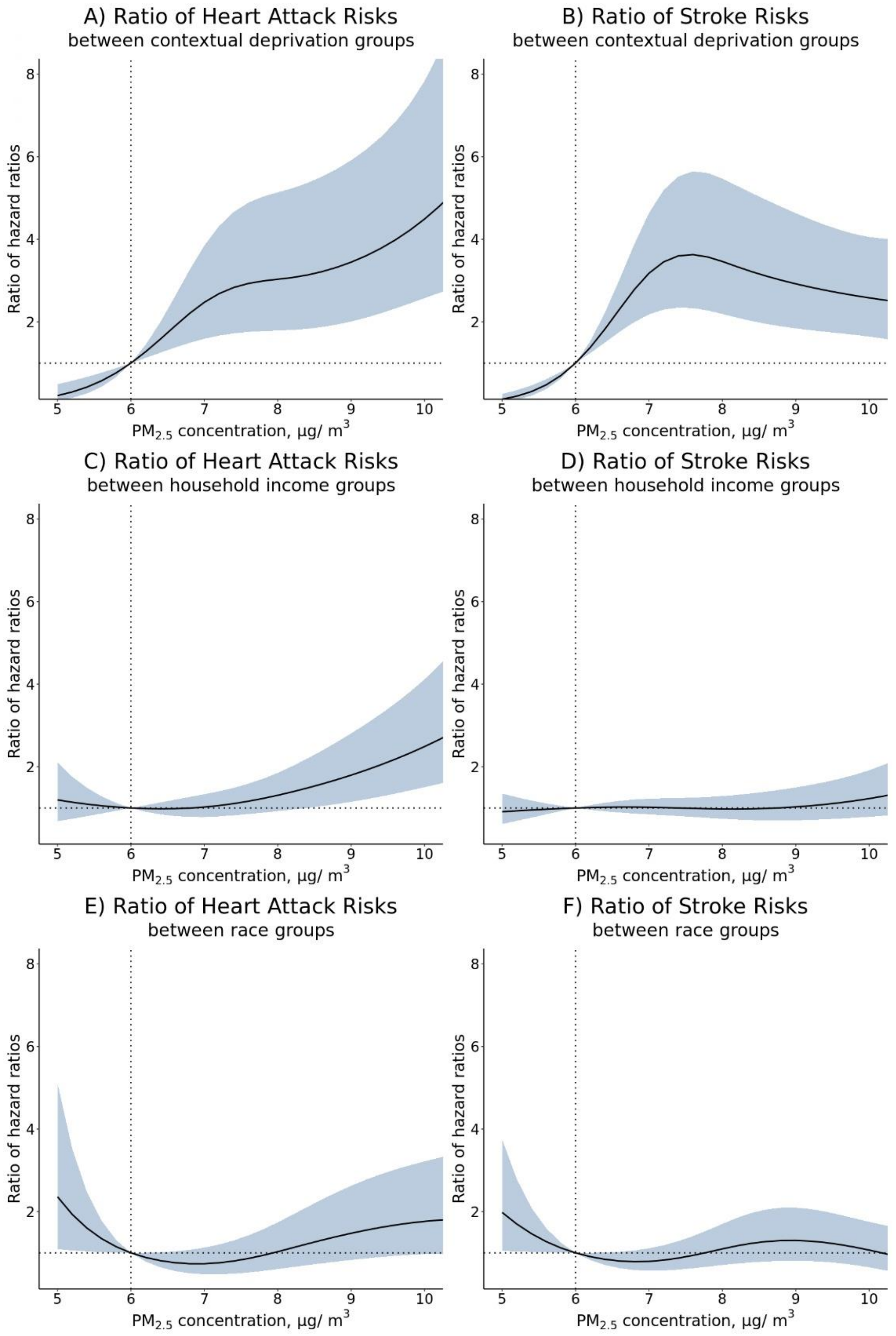

eFigure 18. Ratio of hazard ratios for heart attack or stroke between different subpopulation when placing the knots at the tertiles for the penalized splines.

© 2024 Luo J et al. *JAMA Network Open.*
