# Supplementary material for: Contextual Deprivation, Race and Ethnicity, and Income in Air Pollution and Cardiovascular Disease
Source: JAMA Netw Open. 2024 Aug 19;7(8):e2429137. doi: 10.1001/jamanetworkopen.2024.29137 (PMC11333981; doi:10.1001/jamanetworkopen.2024.29137)
Supplement: Supplement 2. — Data Sharing Statement [file jamanetwopen-e2429137-s002.pdf]

Data Sharing Statement

Luo. Contextual Deprivation, Race and Ethnicity, and Income in Air Pollution and Cardiovascular Disease. *JAMA Netw Open*. Published August 19, 2024. doi:10.1001/jamanetworkopen.2024.29137

Data
Data available: Yes
Data types: Deidentified participant data
How to access data: All data can be accessed on the All of Us researcher workbench: https://www.researchallofus.org/register/
When available: With publication

Supporting Documents
Document types: Statistical/analytic code
How to access documents: All codes can be accessed on the All of Us researcher workbench: https://www.researchallofus.org/register/
When available: With publication

Additional Information
Who can access the data: Anyone who is approved by the All of Us advisory committee
Types of analyses: All analyses can be accessed on the All of Us researcher workbench: https://www.researchallofus.org/register/
Mechanisms of data availability: After approval by All of Us advisory committee
